# Supplementary material for: Ubiquitin Chloromethylketone Probe Enables Activity-Based Protein Profiling of E2 Ubiquitin Conjugating Enzymes
Source: J Am Chem Soc. 2026 Sep 7;148(38):40834–46. doi: 10.1021/jacs.6c08586 (PMC13632588; doi:10.1021/jacs.6c08586)
Supplement: Supplementary file 1 [file ja6c08586_si_002.pdf]

Supplementary Information for

Ubiquitin Chloromethylketone Probe Enables Activity-based Protein Profiling of E2 Ubiquitin Conjugating Enzymes

Saibal Chanda[1], Alan Pham[2], Yugendar R. Alugubelli[2], Nathan Axtell[1], SreeNidhi Karnati[2], Yashwanth Aravapalli[4], James X. Mao[3], Wenyue Cao[2], and Wenshe Ray Liu[1,2,4,5,6*]

[1]Department of Biochemistry and Biophysics, College of Agriculture and Life Sciences, Texas A&M University, College Station, TX 77843

[2]Texas A&M Drug Discovery Center and Department of Chemistry, College of Arts and Sciences, Texas A&M University, College Station, TX 77843

[3]Texas A&M High-Performance Research Computing, Texas A&M University, College Station, TX 77843, USA

[4]Department of Cell Biology and Genetics, School of Medicine, Texas A&M University, College Station, TX 77843

[5]Department of Pharmaceutical Sciences, Irma Lerma Rangel College of Pharmacy, Texas A&M University, College Station, TX 77843, USA

[6]Institute of Biosciences and Technology and Department of Translational Medical Sciences, School of Medicine, Texas A&M University, Houston, TX 77030

*Correspondence should be addressed to Wenshe Ray Liu: wsliu2007@tamu.edu

Materials and Methods

Compounds. TCEP and NTCB were purchased from A2B chem LLC, San Diego, CA, USA. Propargylamine was purchased from TCI Chemicals, America.

Expression and Purification of All Ub Proteins. All Ub proteins including $Ub_{1\text{-}75}$-Cys-6×His, and FLAG-$Ub_{1\text{-}75}$-Cys-6×His were recombinantly expressed and purified. An overnight culture of *E. coli* BL21(DE3) cells harboring a pETDuet-1 expression vector coding $Ub_{1\text{-}75}$-Cys-6×His, or FLAG-$Ub_{1\text{-}75}$-Cys-6×His was inoculated (1:100 dilution) into a 2xYT medium containing 100 µg/mL ampicillin. Cells were let grow at 37 ºC until OD_{600} reached 0.6-0.9 and 1 mM IPTG was added to induce protein expression. The cells were let grow in a shaker at 37 ºC for 8 h or 18 ºC for 16 h and collected by centrifugation (5,000 rpm, 30 min, 4 ºC). The cell pellets were resuspended in a 100 mL lysis buffer (50 mM NaH_2PO_4, 500 mM NaCl, 5 mM imidazole, 1 mM TCEP, pH 7.8) and lysed by sonication. The cell lysates were clarified by centrifugation (10,000 rpm, 30 min, 4 ºC) and the supernatant was decanted. 6 M HCl solution was then added dropwise into the supernatant to adjust pH to 2 to drive the formation of white precipitate inside the solution. The precipitate was pelleted by centrifugation (10,000 rpm, 30 min, 4 ºC) and removed. The pH value of the supernatant was then adjusted back to 7.8 by adding 6 M NaOH. This supernatant was loaded onto high-affinity Ni^{2+}-NTA resins at RT and then washed with a buffer (50 mM NaH_2PO_4, 500 mM NaCl, 30 mM imidazole, 1 mM TCEP, pH 7.8). The bound protein was eluted by another buffer (50 mM NaH_2PO_4, 250 mM NaCl, 300 mM imidazole, 1 mM TCEP, pH 7.8) and then desalted into a 50 mM ammonium bicarbonate (ABC) buffer using HiPrep 26/10 Desalting column (Cytiva). All proteins were concentrated using 3 kDa MWCO Amicon ultracentrifugation filter devices (Millipore Sigma) and concentrations were measured using a NanoDrop spectrophotometer (ThermoFisher). Eventually, all proteins were aliquoted, lyophilized, and stored at -80 ºC.

DFT computation: All density functional theory (DFT) calculations were performed using the Gaussian 16 program, utilizing the advanced computing resources provided by Texas A&M High Performance Research Computing. Molecular geometries were optimized at the M06-2X[2]/6-311+G(d,p) level of theory, followed by frequency analyses to confirm that the structures correspond to true energy minima (no imaginary frequencies) or transition states (one imaginary frequency). Fukui indexes were calculated using NBO charges with NBO 7.0 program. All calculations employed the polarizable continuum model (PCM) with water as the solvent. Calculations were also repeated at the B3LYP[7]-D3BJ[8]/6311G(d,p) level to ensure consistency between methods.

Use of ACPL to Synthesize FLAGUbPA, FLAGUbCMK, and FLAGUbFMK Probes. A 500 mM TCEP stock solution was prepared in water, and a 500 mM NTCB stock solution in DMSO. Solutions of Propargylamine (PA) was made in water at 1 M, while a 1 M solution of as 3-chloro-2,2-diethoxypropan-1-amine and 3-fluoro-2,2-diethoxypropan-1-amine was prepared in DMSO.

Synthesis of FLAGUbCMK, FLAGUbFMK and UbCMK. FLAG-$Ub_{1\text{-}75}$-Cys-6×His protein pellets were dissolved in water, followed by the addition of 3-chloro-2,2-diethoxypropan-1-amine and 3-fluoro-2,2-diethoxypropan-1-amine (final concentration 250mM), TCEP (5 mM), and NTCB (5 mM). The pH was adjusted to 8 and the mixture was incubated at 37 °C. The reaction was desalted using a HiTrap column, then incubated with Ni^{2+}-NTA resins and were removed with micro syringe filters, and the flowthroughs were collected for ESI-MS analysis. Post successful synthesis of both Ub conjugates, trifluoroacetic acid were added and incubated for an hour at RT to deprotect the ethoxy group to afford FLAGUbCMK and FLAGUbFMK respectively. Both the solutions were neutralized using 1M Tris, pH 8.5, purified and collected for further ESI-MS.

To synthesize UbCMK, above steps were followed using $Ub_{1\text{-}75}$-Cys-6×His protein pellets.

Synthesis of FLAGUbPA and UbPA. FLAG-$Ub_{1\text{-}75}$-Cys-6×His and $Ub_{1\text{-}75}$-Cys-6×His protein pellets were dissolved in 500 mM propargylamine, with TCEP and NTCB added (5 mM each). The pH was adjusted to 9 and the mixtures were incubated at 37 °C. After desalting with a HiTrap column, the products were incubated with Ni^{2+}-NTA resins and were removed with micro syringe filters, and the flowthroughs were collected for ESI-MS analysis.

Covalent Capture of DUBs by FlagUbPA and Ub Halomethylketone Probes. 0.1µg/µL UCHL1 (UBP bio), UCHL3 (UBP bio), USP2 CD (UBP bio), USP5 (Enzo), USP7 CD (MCE), GST-USP9X CD (UBP bio), USP10 (R&D system), USP14 (UBP bio), GST-Ataxin-3 (UBP bio), OTUD6B (UBP bio), and 0.1µg/µL FLAGUbPA and two synthesized Ub ketone probes were mixed, and the final reaction volume was adjusted into a 20 µL reaction buffer (50 mM HEPES, 100 mM NaCl, 2 mM TCEP pH 7.85). All reactions were incubated at 37 ºC for 40 min and quenched by adding a 6× SDS loading dye containing 30% β-mercaptoethanol. The reaction mixtures were then analyzed by SDS-PAGE (4-12% bis-tris gel) and Western blotting using HRP-conjugated anti-FLAG.

Covalent capture of E2 enzymes by Ub chloromethyl Ketone probe. 0.1µg/µL UBE2C (UBP bio), UBE2D2 (UBP bio), UBE2D3 (UBP bio), UBE2E1 (UBP bio), UBE2E3 (UBP bio), UBE2I (UBP bio),

UBE2K (UBP bio), UBE2L3 (UBP bio), UBE2L5 (UBP bio), UBE2L6 (UBP bio), UBE2M (UBP bio), UbE2N (UBP bio), UBE2T (UBP bio), UBE2Z (UBP bio), and 0.1µg/µL FlagUbCMK were mixed, and the final reaction volume was adjusted into a 20 µL reaction buffer (50 mM HEPES, 100 mM NaCl, 2 mM TCEP pH 7.85). All reactions were incubated at 37 ºC for 1 h and quenched by adding a 6× SDS loading dye containing 30% β-mercaptoethanol. The reaction mixtures were then analyzed by SDS-PAGE (8-16% bis-tris gel) and Western blotting using HRP-conjugated anti-FLAG.

Labeling of E1 Activating Enzyme. E1 labeling experiments were performed in a ubiquitination buffer containing 50 mM HEPES, 100 mM NaCl, 2 mM TCEP (pH 7.85), 0.1 µg/µL UBE1 (UBP bio) and FLAGUbCMK probe. The reaction mixture was incubated at 37 ºC. Reaction was quenched by adding a 6× SDS loading dye containing 30% β-mercaptoethanol. The mixtures were then analyzed by SDS-PAGE (4-12% bis-tris gel) and Western blotting using HRP-conjugated anti-FLAG.

Activity Based Protein Profiling of in the HEK293T Cell Lysate using Ub Probes. HEK293T cells were cultured in DMEM (Gibco) supplemented with 10% fetal bovine serum (FEB, Gibco) and 5% penicillin-streptomycin (Pen-Strep, Sigma; Pen: 10,000 units/Strep: 10 mg/mL). The cells were grown in T75 flasks in a 37 ºC, 5% CO_2 incubator until they reached approximately 90% confluency. Subsequently, the cells were collected in 1× PBS buffer and transferred into 1.5 mL tubes. To lyse the cells, Pierce (ThermoFisher) IP protease-free cell lysis buffer was added to the cell suspension. The mixture was gently shaken under refrigeration for 30 min to ensure cell lysis. After the cell lysis process, the cell lysate was clarified by centrifugation at 14,000 rpm for 30 min at 4 ºC. The resulting supernatant was collected, and the protein concentration was measured by the BCA assay.

Profiling Using two FLAG-Fused Ub Activity-based Probes. HEK293T cell lysates were incubated separately with FlagUbPA and FlagUbCMK probes for 20 h at 4 ºC before they were analyzed using a 3-8% tris acetate gel and subjected to Western blotting using HRP-conjugated anti-FLAG.

Profiling After depleting UbPA-binding Enzymes. In a separate set of profiling experiments, three aliquoted HEK293T cell lysates were initially incubated with UbPA for 12 h at 4 ºC. Afterward, FLAGUbPA and FLAGUbCMK probes were added separately to three different reaction mixtures, and the incubation continued for another 12 h at 4 ºC. Subsequently, all reactions were analyzed using a 4-12% bis-tris gel followed by Western blotting with HRP-conjugated anti-Flag.

Immunoprecipitation. Anti-DYKDDDDK magnetic agarose beads (Pierce, Thermo Scientific) were equilibrated to room temperature and thoroughly mixed. A 50 µL slurry of beads was transferred to a microcentrifuge tube and washed twice with binding buffer (25 mM Tris-HCl pH 7.4, 150 mM NaCl, 1 mM EDTA, 1% NP-40, 5% glycerol). Probe-treated cell lysates were added and incubated at room temperature with mixing. The beads were then washed three times with 1× PBS + 0.01% tween-20 and once with purified water. Bound proteins were acid-eluted, and pH was adjusted with neutralization Buffer. The elution was repeated three times for maximum recovery.

Proteomic Identification of Cysteine Enzymes in HEK293T Cell Lysates. Protein Digestion: Eluted proteins from co-immunoprecipitation were pooled and subsequently lyophilized. Then, the powdered proteins were resuspended in an SDS solution and lysed to ensure full dissolution. Resolubilized proteins were then reduced by 5 mM tris(2-carboxyethyl)phosphine at 55°C for 15 min and then alkylated by 20 mM Methyl Methanethiosulfonate at room temperature for 10 minutes. 27.5% (v/v) Phosphoric Acid was then used in to achieve a final 1% concentration and acidify the samples. These acidified samples were subsequently applied to S-Trap Micro columns (C002) after being quenched with 165 µl 100 mM TEAB in 90% LC-MS grade MeOH at a pH of 7.5. This same TEAB solution was used to then wash the sample five times while it resided on the column, each consisting of 225 µl. Samples were then digested on-column by a trypsin solution (1 µg trypsin in 20 µl 50 mM TEAB, pH 8.0) for 18 hours at 37°C. The digested proteins, now in peptide form, were subsequently eluted with 40 µl of 50 mM TEAB (pH 8.0), 0.2% formic acid, and 0.2% formic acid in 50% acetonitrile. Samples were then dried and reconstituted using 0.2% formic acid.

Mass Spectrometry Analysis: Digested proteins were analyzed by an Orbitrap Exploris 480 mass spectrometer, which was coupled with a Vanquish Neo UHPLC. The column used was a EASY-Spray™ PepMap™ Neo UHPLC Column (50cm length, 75µm internal diameter, C18 resin). Solvents A (0.1% formic acid in water) and B (0.1% formic acid in 100% acetonitrile) were used in a gradient of 2-40% over 90 minutes at a flow rate of 200 nl/min. Full-scan MS was surveyed from 375-1475 with a mass resolution of 120000 in positive-ion mode. Using a cycle time of three seconds, precursors were selected for MS/MS at a resolution of 15000 and analyzed using a higher energy collision-induced dissociation with 30% normalized collision energy. The isolation window was left at 1.6 m/z, the default charge at 2, the automatic gain control at 50% normalized target, an automatic maximum injection time, and a custom dynamic exclusion (exclude after 1 time for 30 seconds).

Proteome Discoverer: The acquired spectra were analyzed by Proteome Discoverer 3.2 The spectrum files RC node and Minora feature detector node were used in tandem to recalibrate mass accuracy and detect chromatographic peaks respectively. The MS/MS spectra was searched against a reverse-concatenated human protein sequence database using both SEQUEST HT and MS Amanda nodes to ensure maximum amounts of protein were obtained. These matches were then evaluated by Percolator to ensure a low false discovery rate.

The consensus workflow was used to evaluate these results. The feature mapper node was used for chromatographic retention time alignment, with a maximum retention time window of 10 minutes and a mass tolerance of 5 ppm. The peptide abundances were normalized using the total peptide amount method and quantified using the summed abundances method. The ratio between proteins was calculated using the protein abundance-based method, and the hypothesis test used was the ANOVA method to generate p-values and adjusted p-values, used to prioritize candidates. The volcano plot was then generated using two technical replicates (injections of our digest) of our experiment. P-values represent reproducibility of the injections. Candidates were selected based on a $\log2FC \geq 1$, and an adjusted p-value of < 0.20, for further validation.

Inhibition of Ub Transfer by FLAGUbCMK in E1-E2 cascade. Ub transfer from E1 to E2 was monitored in a reconstituted cascade using recombinant human UBE1, UBE2D3, non-FLAG Ub-CMK and FLAG-tagged Ub. The reaction was performed in assay buffer (50 mM Tris-HCl, 100 mM NaCl, 5 mM ATP, 10mM $MgCl_2$, 2 mM TCEP, pH 7.8) at 37 °C. For standard reactions, UBE1 (1 µM), UBE2D3 (3 µM), non-FLAG UbCMK (1 µM) and FLAG-Ub (15 µM) were combined in assay buffer and incubated for 120 min. Reactions were quenched by addition of 4× SDS–PAGE loading buffer containing 30% β-mercaptoethanol. Samples were analyzed by (4-12% bis-tris gel and analyzed by western blotting with an anti-FLAG antibody.

Inducing Physiological Stress in HEK293T Cell Lysate. HEK293T cells were maintained in DMEM supplemented with 10% FBS and 1% penicillin–streptomycin at 37 °C in a humidified incubator with 5% CO_2. For all stress-induction experiments, cells were seeded 24 h prior to treatment to reach ~70–80% confluency at the time of compound addition. Unless otherwise specified, treatments were performed in complete growth medium. Following treatment, cells were immediately placed on ice, washed twice with ice-cold PBS, and processed for lysis as described below.

Oxidative Stress-Diamide: Oxidative/thiol-oxidizing stress was induced using diamide. HEK293T cells were washed once with pre-warmed PBS and incubated with fresh complete medium containing 400 μM diamide (from a freshly prepared aqueous stock) for 30 min at 37 °C.

Proteotoxic Stress-MG132: Proteotoxic stress resulting from proteasome inhibition was induced using MG132. A 10 mM stock solution of MG132 in DMSO was diluted into complete medium to a final concentration of 10 μM. HEK293T cells were incubated with MG132-containing medium for 6 h at 37 °C.

Inflammatory Stress-Poly(I:C): Inflammatory signaling was induced using polyinosinic:polycytidylic acid [poly(I:C)] to activate TLR3-dependent pathways. Poly(I:C) was dissolved in nuclease-free water and added directly to complete medium at a final concentration of 10 μg/mL. HEK293T cells were treated with poly(I:C) for 12 h at 37 °C.

Metabolic Stress-2-Deoxy-D-glucose (2-DG): Metabolic/glycolytic stress was induced by inhibiting glycolysis with 2-deoxy-D-glucose (2-DG). A 1 M stock solution of 2-DG in water was diluted into complete medium to a final concentration of 10 mM. HEK293T cells were incubated with 10 mM 2-DG for 12 h at 37 °C.

Lipid Oxidative Stress-Peroxynitrite: Lipid oxidative stress was modelled using oxidized LDL (oxLDL) generated by peroxynitrite treatment. HEK293T cells were incubated with peroxynitrite (final concentration 400 μM) for 30 mins at 37 °C.

Genotoxic Stress-Etoposide: DNA damage was induced using the topoisomerase II inhibitor etoposide. A 10 mM stock solution of etoposide in DMSO was diluted into complete medium to a final concentration of 20 μM. HEK293T cells were treated with etoposide for 10 h at 37 °C.

Cell Lysis: For all conditions, after the final PBS wash, cells were scraped into ice-cold IP lysis buffer and incubated on ice for 20–30 min with occasional mixing. Lysates were clarified by centrifugation (e.g., 14,000 × g, 10–15 min, 4 °C), and protein concentrations were determined by BCA assay prior to subsequent probe labeling.

Negative Control Treatment: For negative controls of active-site–dependent FLAGUbCMK labeling, HEK293T cells were pretreated with the indicated inhibitors prior to lysis. For global thiol blockade (UBE2D2/UBE2D3), cells were incubated with N-ethylmaleimide (NEM, 5 mM, 1 h, 37 °C) in complete medium, followed by a PBS wash. For UBE2M, UBE2N, and UBE2L3, cells were treated in complete medium with micafungin (50 μM, 6 h), NSC-697923 (5 μM, 6 h), or dimethyl fumarate (DMF, 50 μM, 6 h), respectively, at 37 °C. After inhibitor pretreatment, cells were placed on ice, washed with ice-cold PBS, and processed for lysis as described above.

Synthesis of 3-chloro-2,2-diethoxypropan-1-amine.

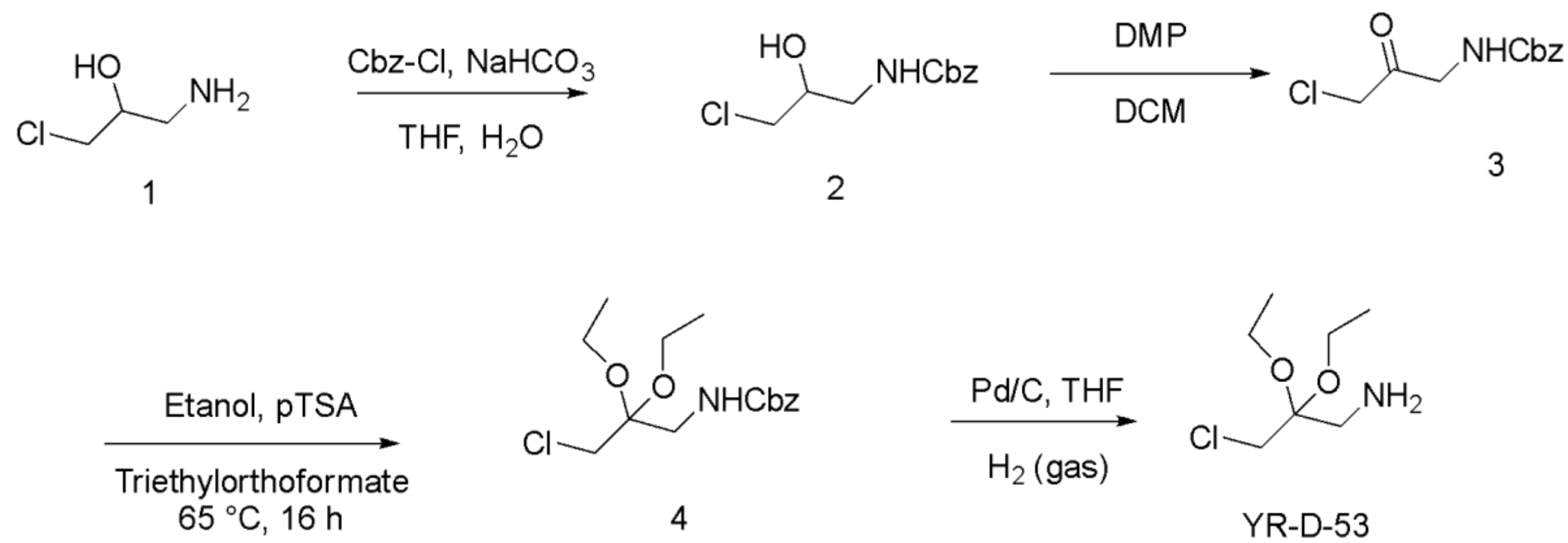

Benzyl (3-chloro-2-hydroxypropyl)carbamate (2): To a mixture of 1 (5.0 g, 34 mmol, 1.0 eq) in THF (50 mL) and H_2O (25 mL), $NaHCO_3$ (11.78 g, 136 mmol, 4.0 eq) was added. And Cbz-Cl (1.2 eq) was added as a dropwise to the reaction mixture. Then the mixture was stirred at rt for 16 h, diluted with EtOAc (100 mL). The organic layer was washed with water (50 mL), dried over sodium sulphate and concentrated under reduced pressure. The residue was purified by silica gel (PE : EA = 7: 3) to give 2 (6 g, yield: 74%) as a yellow oil. ^{1}H NMR (400 MHz, DMSO) δ 7.48 – 7.26 (m, 6H), 5.02 (s, 2H), 3.72 (qd, J = 6.0, 4.2 Hz, 1H), 3.60 (dd, J = 11.2, 4.3 Hz, 1H), 3.49 (dd, J = 11.2, 5.8 Hz, 1H), 3.09 (qt, J = 13.6, 6.0 Hz, 2H). ^{13}C NMR (101 MHz, DMSO) δ 156.78, 137.61, 129.31, 129.11, 128.82, 128.80, 128.25, 128.22, 69.83, 65.80, 48.18, 44.54.

benzyl (3-chloro-2-oxopropyl)carbamate (3): To a solution of 2 (1.0 g, 4.27 mmol) in CH_2Cl_2 (30 mL) was added $NaHCO_3$ (1.47 g, 4 eq) and the Dess-Martin reagent (2.24 g, 5.13 mmol, 1.2 eq). The resulting mixture was stirred at rt for 4 h. Then the reaction was quenched with a saturated $NaHCO_3$ solution containing 10 % $Na_2S_2O_3$. The layers were separated. The organic layer was then washed with saturated brine solution, dried over anhydrous Na_2SO_4 and concentrated on vacuum. The residue was then purified with flash chromatography afford 3 as white solid (750 mg, 75%). ^{1}H NMR (400 MHz, DMSO) δ 7.59 (t, J = 5.8 Hz, ^{1}H), 7.46 – 7.17 (m, 5H), 5.05 (s, 2H), 4.56 (s, 2H), 4.02 (d, J = 5.9 Hz, 2H). ^{13}C NMR (101 MHz, DMSO) δ 199.33, 156.91, 137.41, 128.84, 128.33, 128.22, 66.09, 48.38, 47.66.

Benzyl (3-chloro-2,2-diethoxypropyl)carbamate (4): To a stirred solution of 3 (700 mg, 3.02 mmol), triethyl orthoformate (1 mL, 6.04 mmol) and TsOH (cat.) were heated to reflux in ethanol (20mL) with Dean Stark trap overnight. After cooled down to rt and diluted with ethyl acetate, the resulting solution was washed with $NaHCO_3$ (sat) and brine. The organic later was dried over $MgSO_4$, filtered and evaporated. The crude product was purified by column chromatography (silica gel, 0 to 40% ethyl acetate in hexane), get the compound 4 (500 mg, yield: 55%) as an oil. ^{1}H NMR (400 MHz, DMSO) δ 7.52 – 7.17 (m, 6H), 5.05 (s, 2H), 3.61 (s, 2H), 3.55 – 3.37 (m, 4H), 3.29 (d, J = 6.3 Hz, 2H), 1.09 (t, J = 7.0 Hz, 6H).

3-chloro-2,2-diethoxypropan-1-amine (YR-D-53): To a solution of 4 (500 mg, 1.59 mmol,) in THF (30 mL) was added 10 % Pd/C (0.1 eq). The mixture was then stirred with hydrogen balloon at room temperature for 16 h. The catalyst was then filtered off, and the solution was evaporated on vacuo to afford the final compound as gummy solid (quant.) ^{1}H NMR (400 MHz, $CDCl_3$) δ 3.61 (s, 2H), 3.57 – 3.48 (m, 4H), 2.92 (s, 2H), 1.20 (t, J = 7.0 Hz, 6H). ^{13}C NMR (101 MHz, $CDCl_3$) δ 101.87, 56.32, 41.96, 41.86, 15.25. HRMS- ESI (m/z) [M+H]+ calc'd for $C_7H_{17}ClNO_2$+, 182.0928, found 182.0942.

Synthesis of 3-fluoro-2,2-diethoxypropan-1-amine.

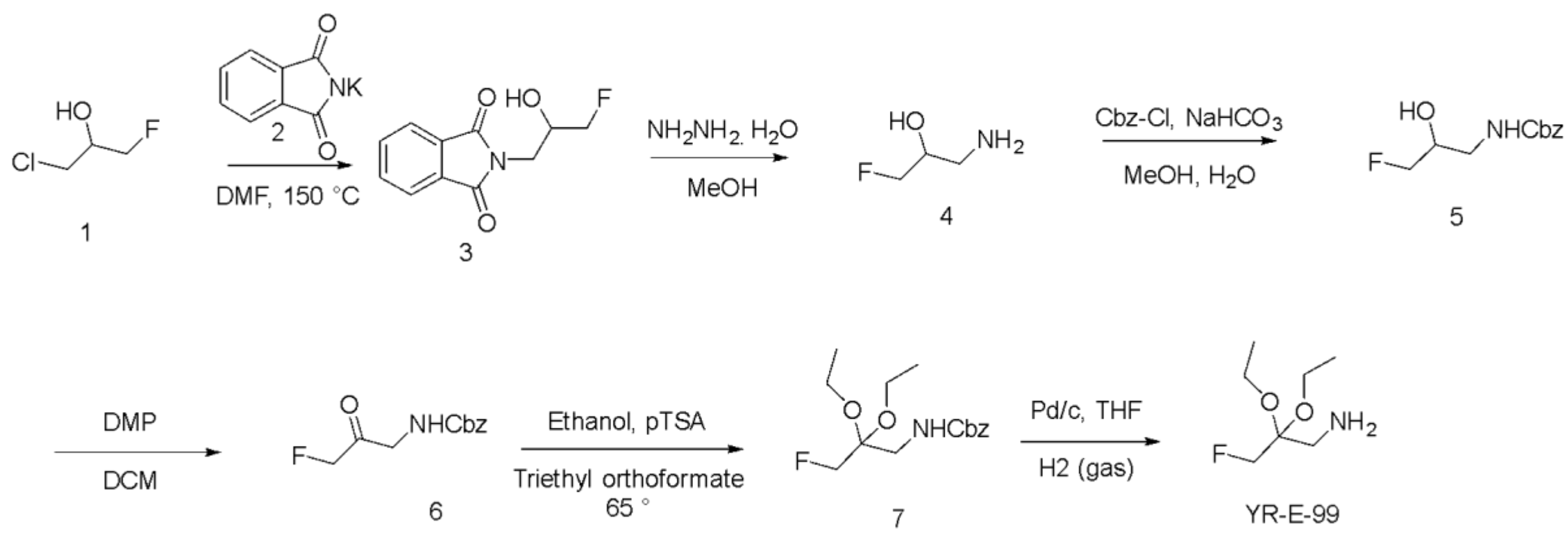

2-(3-fluoro-2-hydroxypropyl)isoindoline-1,3-dione (3): To a suspension solution of potassium phthalimide (8.26 g, 44.64 mmol) in DMF (30 mL) was added 1-chloro-3-fluoro-propan-2-ol (5 g, 44.64 mmol), and the mixture was stirred at 130 °C for 4 h. After cooling, the mixture was diluted with EtOAc (200 mL) and washed with water (50 mL). The organic layer was separated and concentrated under vacuum. The residue was purified by column chromatography to give S1 (8 g, 80% yield). 1H NMR (400 MHz, DMSO) δ 7.93 – 7.81 (m, 4H), 5.41 (d, J = 4.7 Hz, 1H), 4.39 (dddd, J = 47.5, 27.1, 9.7, 4.6 Hz, 2H), 4.03 (q, J = 7.1 Hz, 1H), 3.62 (qd, J = 13.8, 6.6 Hz, 2H).

benzyl (3-fluoro-2-hydroxypropyl)carbamate (5): Add hydrazine hydrate (0.95 eq) to a solution of 2-(3-fluoro-2-hydroxypropyl)isoindoline-1,3-dione (8.0 g) in MeOH (300 mL). Stir the reaction mixture at ambient temperature for 20 hours, during which time a precipitate form. Add concentrated aqueous HCl (1 mL) to the mixture. Stir the reaction mixture for 3 hours. Remove the precipitate by filtration. Remove the MeOH in vacuo. Take crude compound as mixture of 4 (1.0 eq) in THF (100 mL) and H_2O (50 mL), $NaHCO_3$ (4.0 eq) was added. And Cbz-Cl (1.2 eq) was added as a dropwise to the reaction mixture. Then the mixture was stirred at rt for 16 h, diluted with EtOAc (100 mL). The organic layer was washed with water (50 mL), dried over sodium sulphate and concentrated under reduced pressure. The residue was purified by silica gel (PE : EA = 7: 3) to give 5 (yield: 60%) as a yellow solid.

benzyl (2,2-diethoxy-3-fluoropropyl)carbamate (7): To a solution of 5 (4.0 g, 17.6 mmol) in CH_2Cl_2 (60 mL) was added $NaHCO_3$ (6.06 g, 4 eq) and the Dess-Martin reagent (8.98 g, 21.12 mmol, 1.2 eq). The

resulting mixture was stirred at rt for 4 h. Then the reaction was quenched with a saturated $NaHCO_3$ solution containing 10 % $Na_2S_2O_3$. The layers were separated. The organic layer was then washed with saturated brine solution, dried over anhydrous Na_2SO_4 and concentrated on vacuum. The residue was then purified with flash chromatography afford 3 as white solid. To a stirred solution of 6 (2.5 g, 11.11 mmol), triethyl orthoformate (3.71 mL, 22.22 mmol) and TsOH (cat.) were heated to reflux in ethanol (30 mL) overnight. After cooled down to rt and diluted with ethyl acetate, the resulting solution was washed with $NaHCO_3$ (sat) and brine. The organic later was dried over $MgSO_4$, filtered and evaporated. The crude product was purified by column chromatography (silica gel, 0 to 40% ethyl acetate in hexane), get the compound 7 (1.85 g, yield: 55%) as an oil. ^{1}H NMR (400 MHz, $CDCl_3$) δ 7.41 – 7.27 (m, 5H), 5.11 (s, 2H), 4.90 (d, J = 6.5 Hz, 1H), 4.38 (s, 1H), 4.26 (s, 1H), 3.62 – 3.45 (m, 6H), 1.18 (t, J = 7.0 Hz, 6H).

2,2-diethoxy-3-fluoropropan-1-amine (YR-E-99): To a solution of 7 (1.8 g, 6.02 mmol,) in THF (50 mL) was added 10 % Pd/C (0.1 eq). The mixture was then stirred with hydrogen balloon at room temperature for 16 h. The catalyst was then filtered off, and the solution was evaporated on vacuo to afford YR-E-99 as gummy solid (quant.). ^{1}H NMR (400 MHz, DMSO) δ 4.44 (s, 1H), 4.33 (s, 1H), 3.47 (qd, J = 7.0, 2.0 Hz, 4H), 2.64 (d, J = 2.5 Hz, 2H), 1.11 (t, J = 7.1 Hz, 6H). ^{13}C NMR (101 MHz, DMSO) δ 100.72, 100.51, 79.65, 77.95, 49.06, 42.05, 15.73.

Acknowledgments

This work was supported by the Welch Foundation (grant A-1715 to W.R.L.) and National Institutes of Health (grants R35GM145351 to W.R.L., R01GM129076). The authors thank Mr. Yohannes Rezenom in the Mass Spectrometry Facility of Texas A&M University for helping with running the LC-MS characterizations of all proteins.

Supplementary Figures

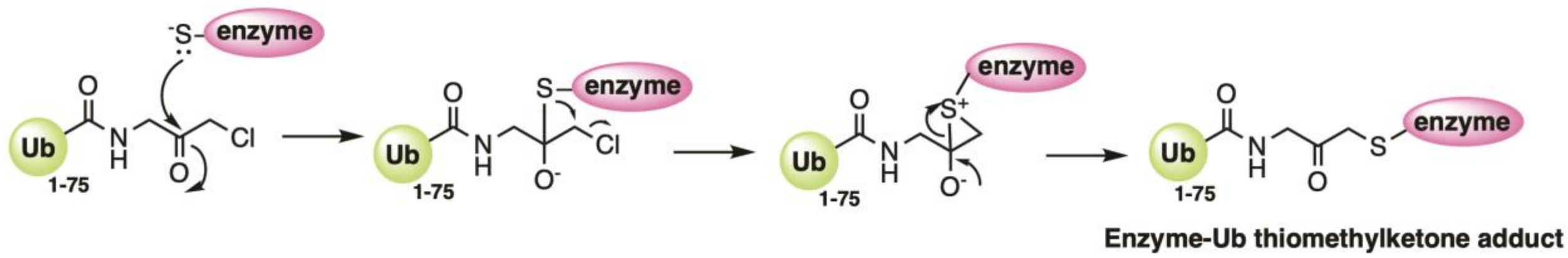

Figure S1. Reaction mechanism for UbCMK with a cysteine enzyme to form an enzyme-Ub thiomethylketone adduct.

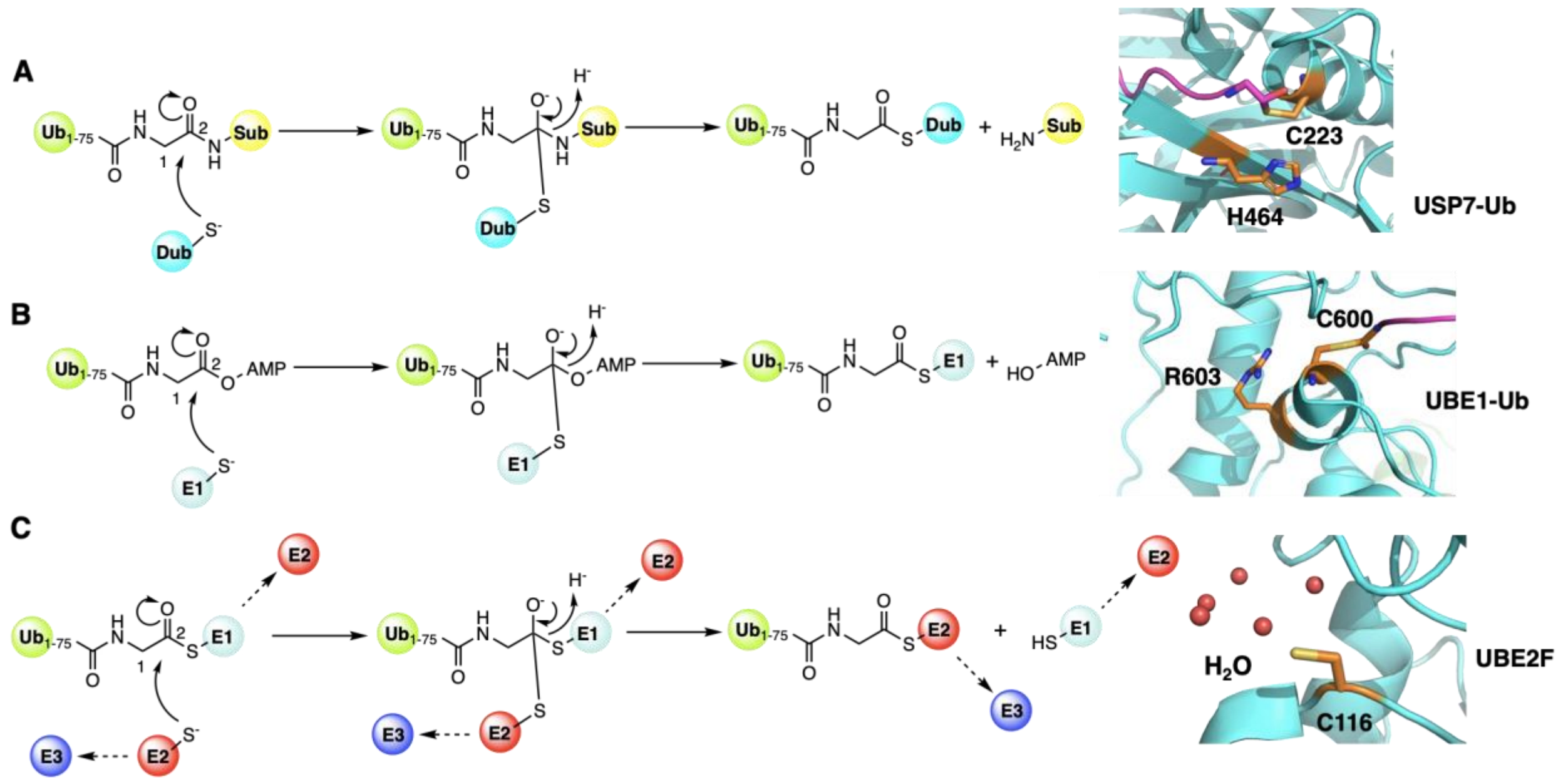

Figure S2. Canonical mechanisms of catalysis for Deubiquitinases (DUBs), E1 activating enzymes, and E2 conjugating enzymes, with representative crystal structures. (A) Cysteine DUBs such as USP7 (PDB: 5JTV) employ an active-site cysteine (C223) that is strongly activated by a catalytic histidine (H464), giving a low pKa, highly nucleophilic thiolate that efficiently attacks the electrophilic isopeptide carbonyl between Ub and substrate; the USP7-Ub complex highlights this tightly organized catalytic dyad. (B) Ub E1 enzymes (e.g., UBE1) use an active-site cysteine (C600) whose thiolate (PDB: 6DC6) is strongly activated and maintained at low pKa by nearby basic residues, including R603, enabling a highly nucleophilic attack on the Ub-adenylate intermediate in which AMP both enhances electrophilicity. (C) In contrast, the E2 conjugating enzyme UBE2F (PDB: 3FN1) contains an active-site cysteine (C116) that is relatively solvent exposed, corresponding to a higher pKa and weaker nucleophilicity; this less activated thiolate participates in a thermodynamically modest thioester exchange with E1-linked Ub, making forward transfer less favourable than the DUB and E1 cysteine reactions.

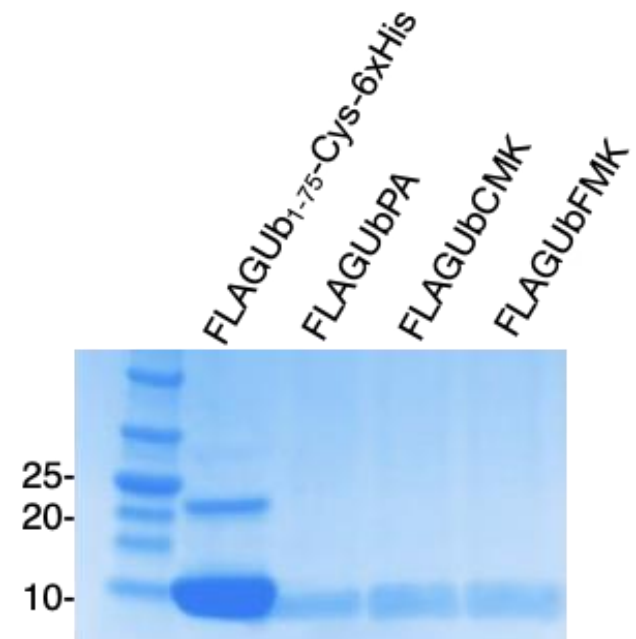

Figure S3. SDS-PAGE and Coomassie blue staining of FLAG-Ub_{1-75}-Cys-6×His, and synthesized probes FLAGUbPA, FLAGUbCMK, FLAGUbFMK

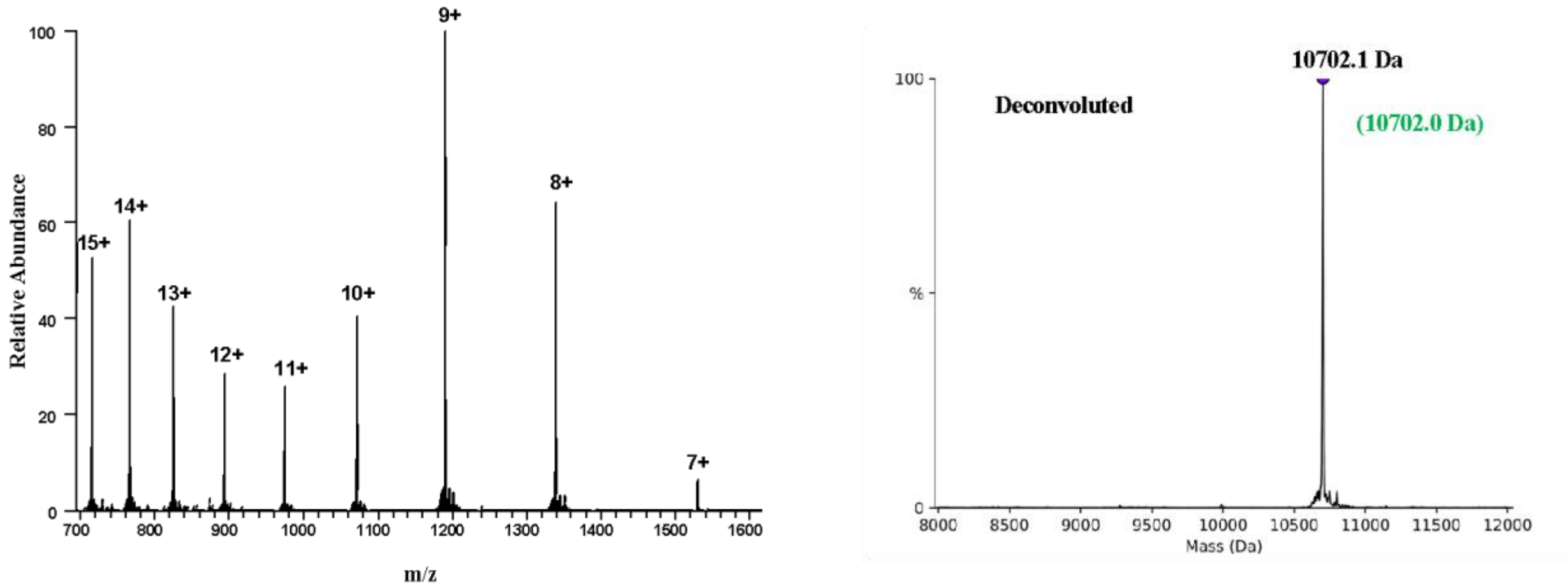

Figure S4. ESI-MS spectra and deconvoluted mass of FLAG-Ub$_{1\text{-}75}$-Cys-6×His. The value in green indicates the actual theoretical mass of the protein.

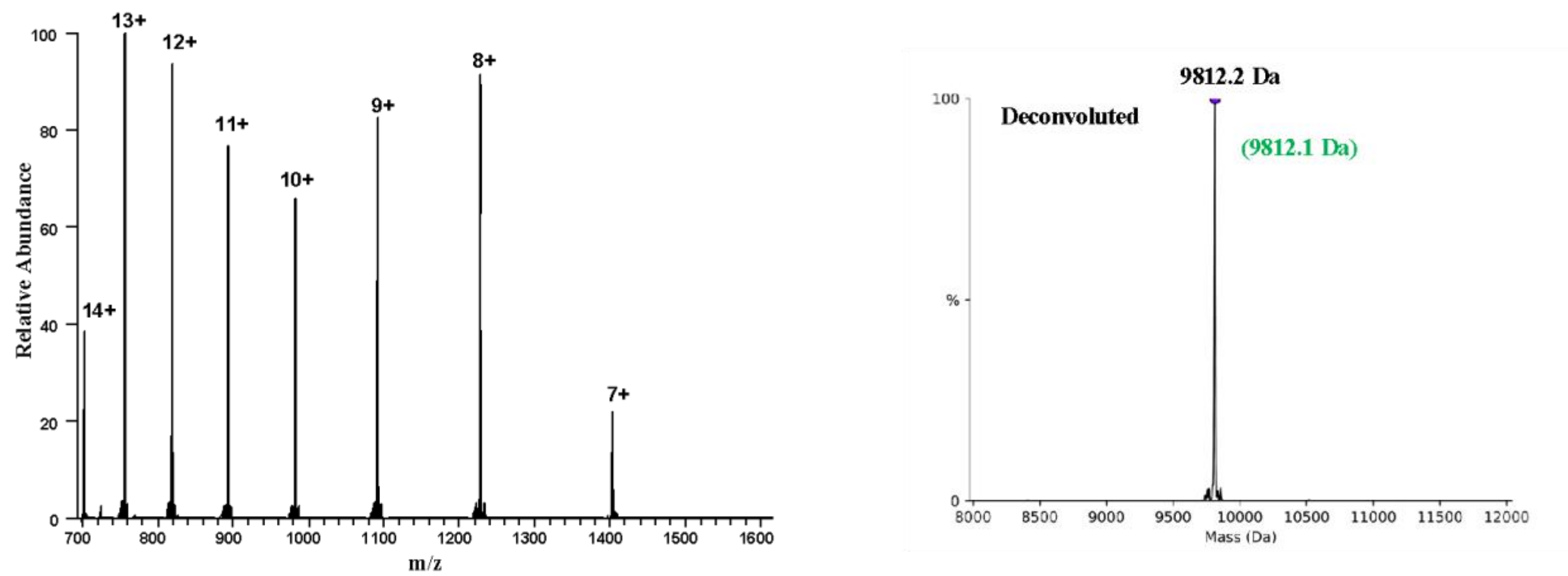

Figure S5. ESI-MS spectra and deconvoluted mass of FLAGUbPA. The value in green indicates the actual theoretical mass of the probe.

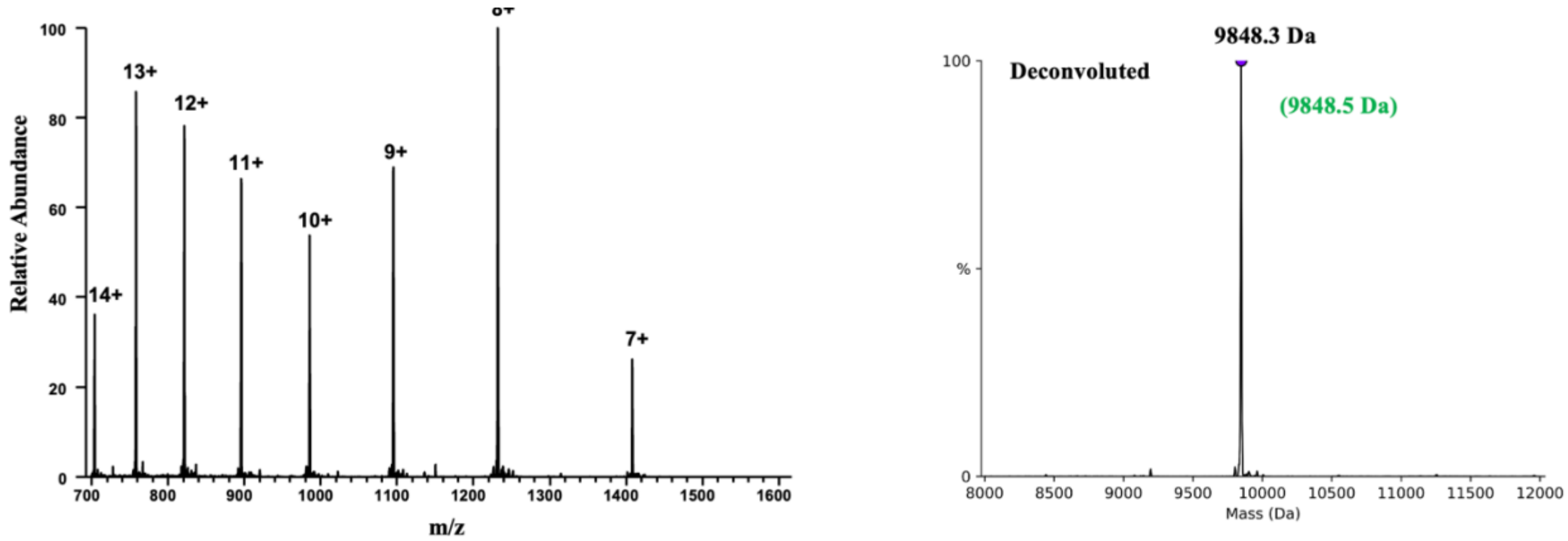

Figure S6. ESI-MS spectra and deconvoluted mass of FLAGUbFMK. The value in green indicates the actual theoretical mass of the probe.

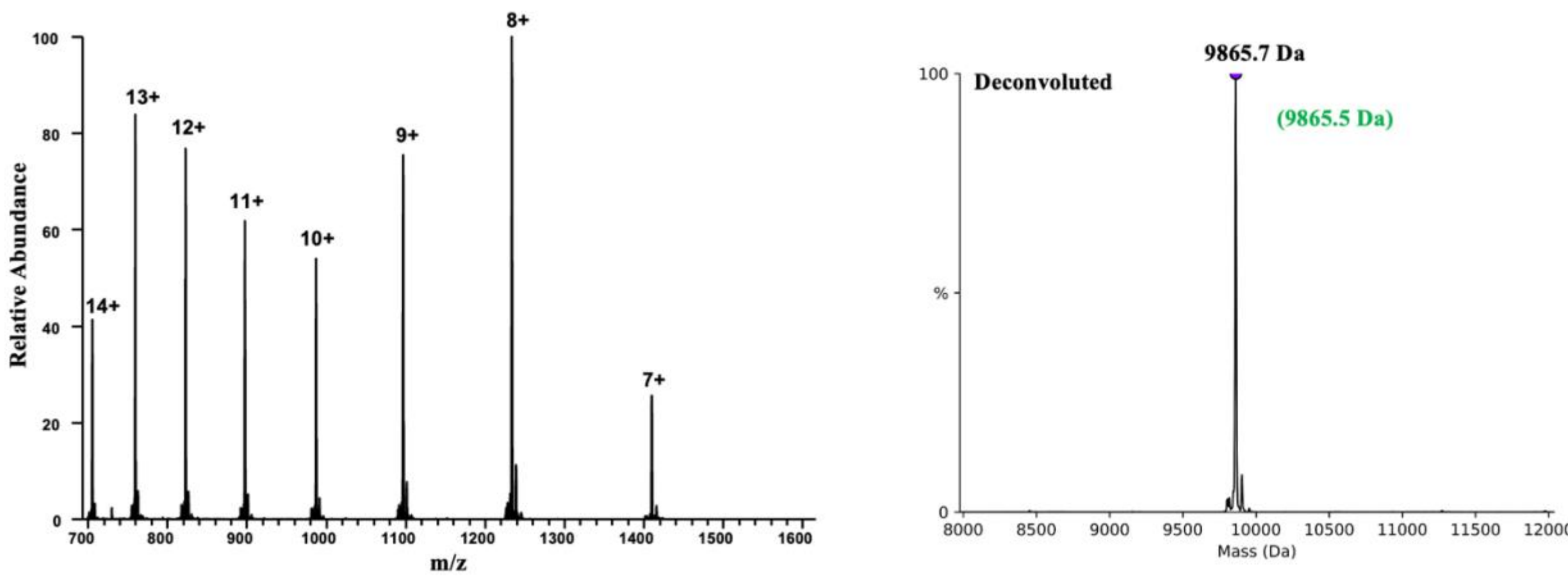

Figure S7. ESI-MS spectra and deconvoluted mass of FLAGUbCMK. The value in green indicates the actual theoretical mass of the probe.

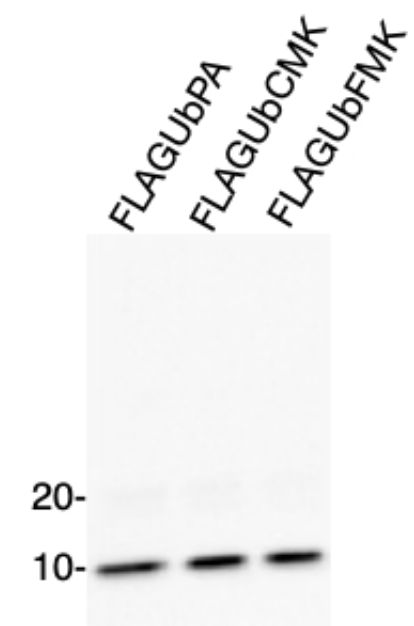

Figure S8. Anti-Flag western blot detection for the synthesized FLAG fused Ub probes to confirm that Flag antibody is able to bind to the Flag epitope of the probes.

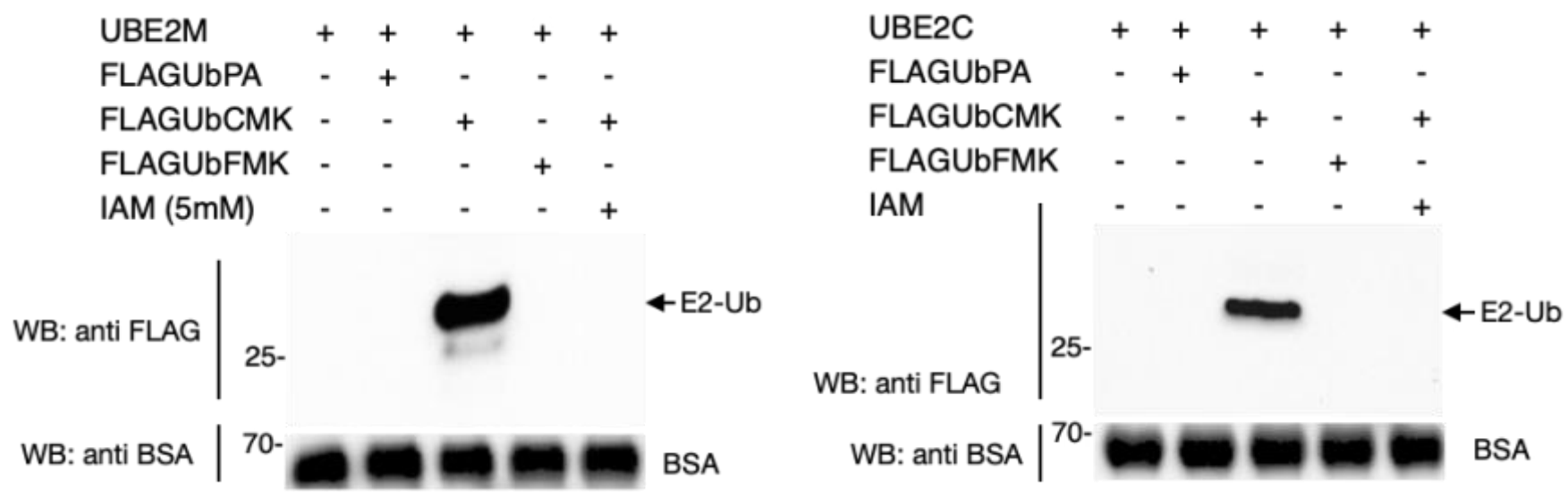

Figure S9. Use of BSA loading control in the reaction of the Ub probes with UBE2M and UBE2C. The probes and the enzymes were reconstituted in a reaction buffer containing BSA. The BSA was detected using anti-BSA antibody, it was used as a loading control for the reaction.

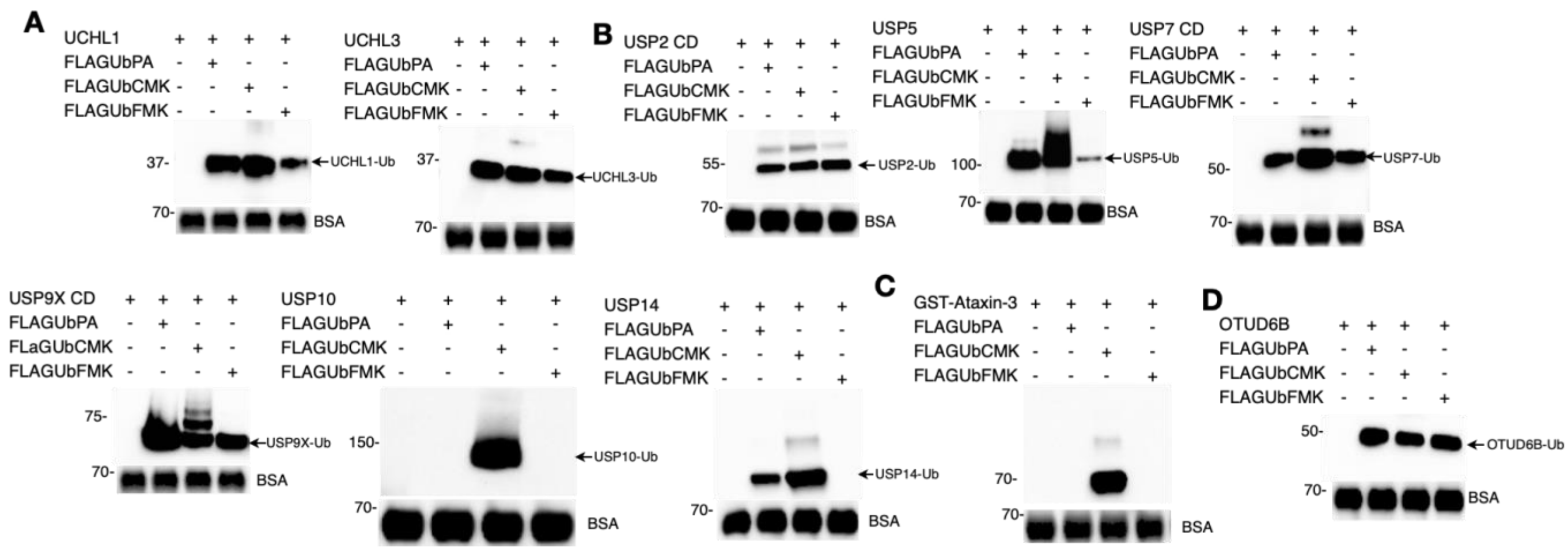

Figure S10: Reaction between the three Ub probes and DUBs from different superfamily (A) UCH superfamily (B) USP superfamily (C) Josephin superfamily (D) OTU superfamily. The probes and the enzymes were reconstituted in a reaction buffer containing BSA. The BSA was detected using anti-BSA antibody, it was used as a loading control for the reaction. SDS–PAGE/anti-Flag immunoblots of recombinant DUBs incubated with FLAGUbPA, FLAGUbFMK, or FLAGUbCMK under identical conditions. Distinct higher–molecular-weight bands corresponding to Ub–DUB adducts are observed for most enzymes. For all tested DUBs, FlagUbCMK exhibits labeling comparable to or greater than FlagUbPA. FlagUbFMK shows weaker and more restricted reactivity: it labels USP5 with markedly lower intensity than FlagUbPA and fails to label USP10, USP14, or Ataxin-3 under conditions where FLAGUbPA gives clear signals for USP5, and USP14 (and where neither probe labels USP10 and Ataxin-3).

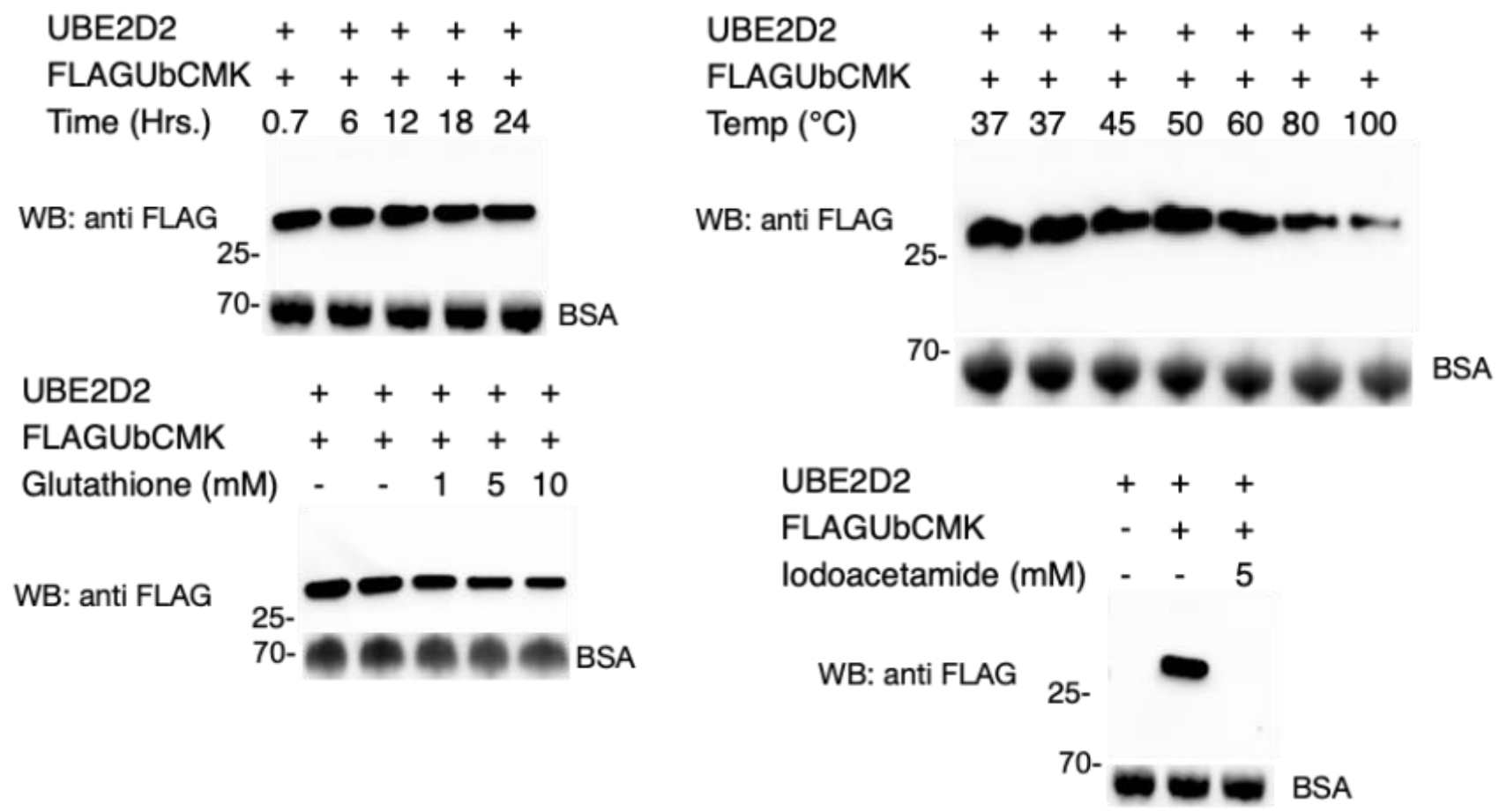

Figure S11. Use of BSA loading control in the characterization of UBE2D2-FLAGUbCMK thioether complex. (**A**) Time dependent assay (**B**) Stability of the complex over time (**C**) Thiol exchange assay **(D)** Cysteine labelling experiment. BSA was used as a loading control.

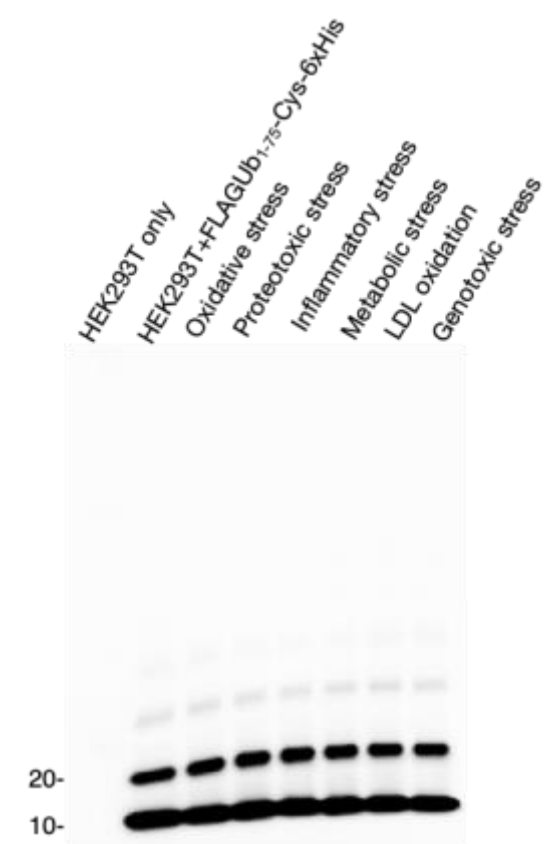

Figure S12: Negative control experiments in which HEK293T cell lysates at various stages (regular activity-based profiling and stress-based study) of the study were incubated with recombinant FLAG-$Ub_{1\text{-}75}$-Cys-6×His. No labeling of any cysteine enzymes were observed after anti-FLAG western blot detection.

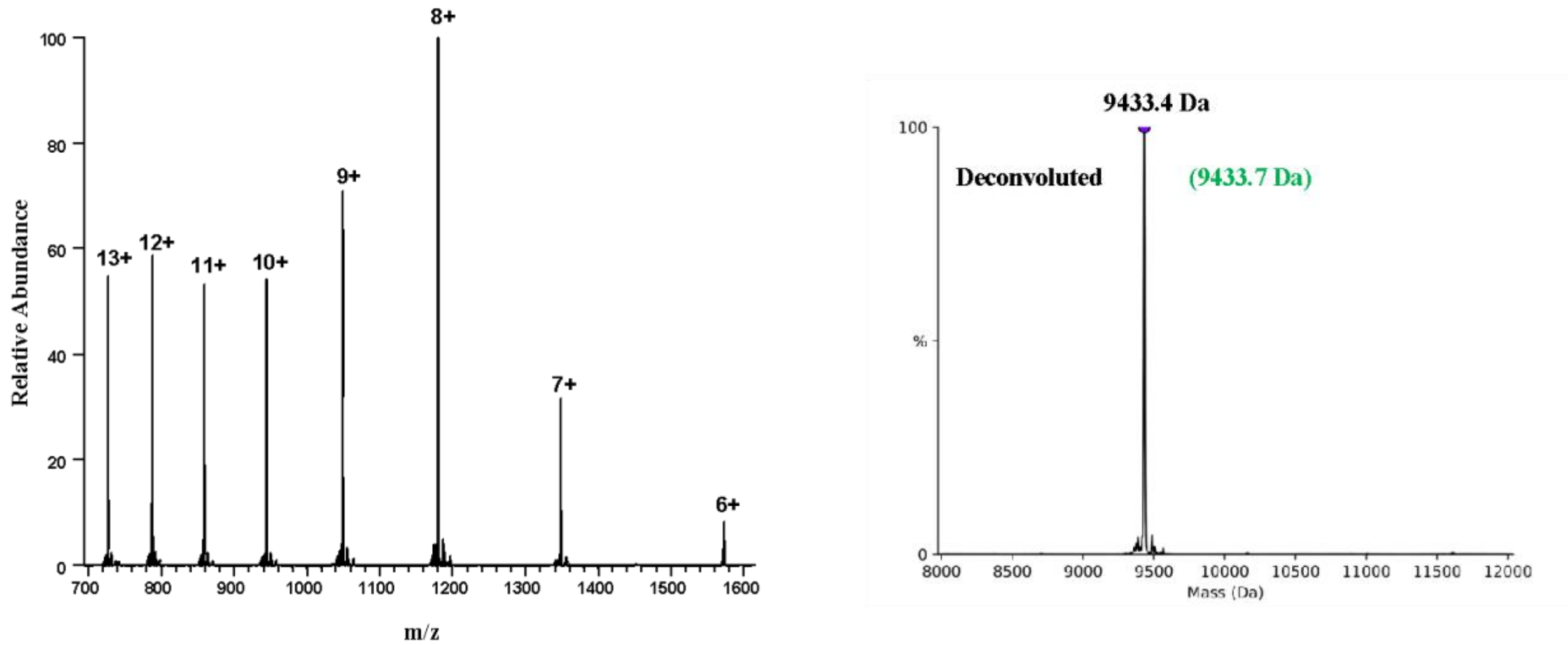

Figure S13. ESI-MS spectra and deconvoluted mass of Ub$_{1-75}$-Cys-6×His. The value in green indicates the actual theoretical mass of the protein.

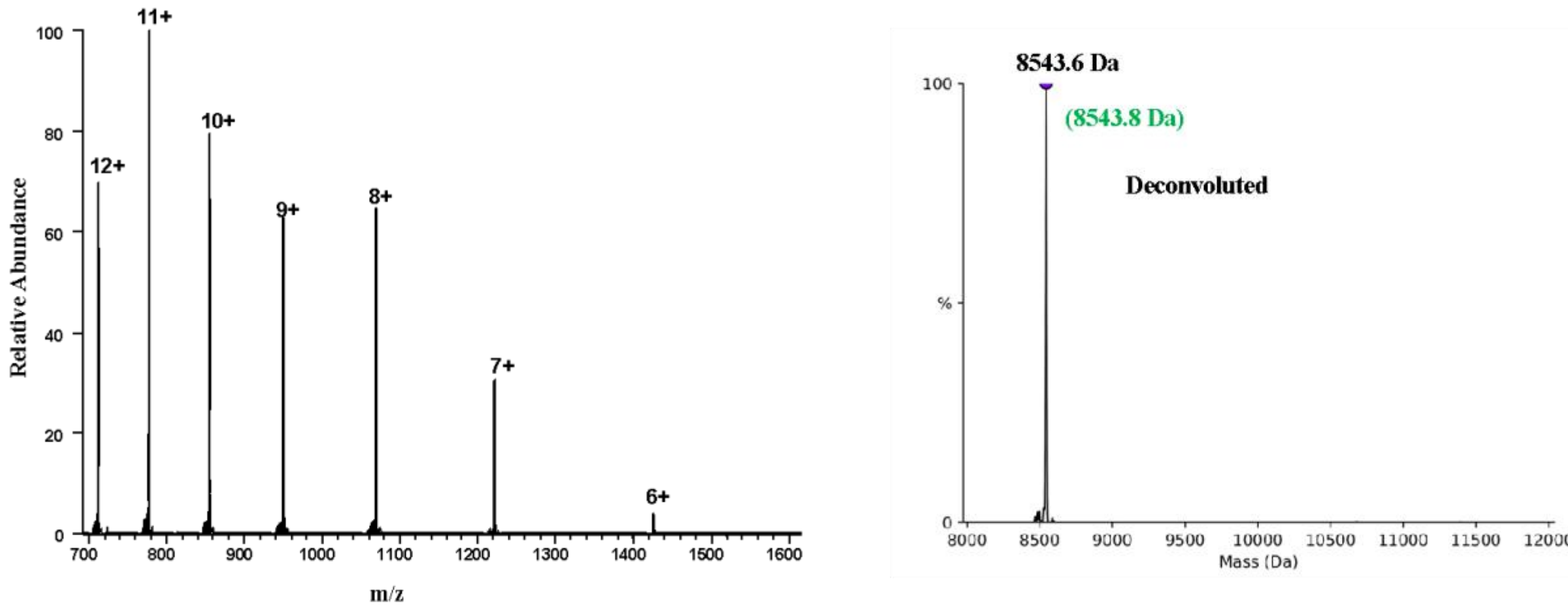

Figure S14. ESI-MS spectra and deconvoluted mass of UbPA. The value in green indicates the actual theoretical mass of the probe.

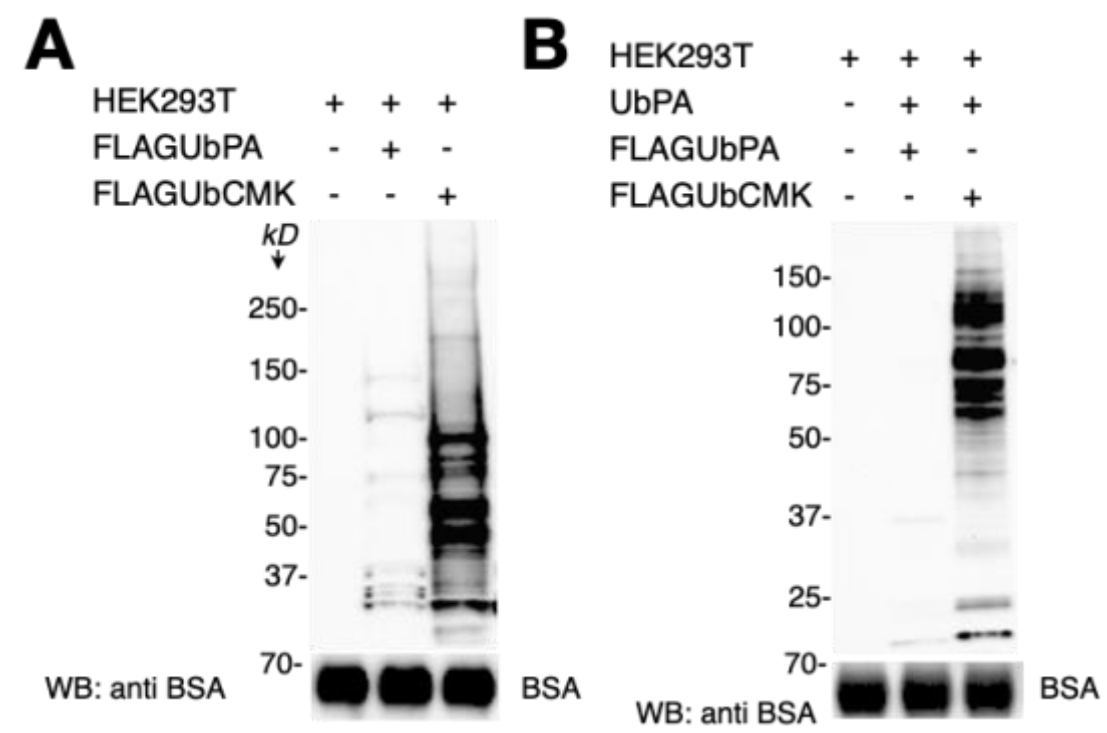

Figure S15. BSA loading control in profiling the cysteine enzymes in HEK293T cell lysates. (**A**) The whole cell lysates treated with FLAGUbPA and FLAGUbCMK probes. (**B**) The whole cell lysates pretreated with UbPA followed by addition of the FLAGUbCMK probe. The BSA was detected using anti-BSA antibody, it was used as a loading control for the reaction.

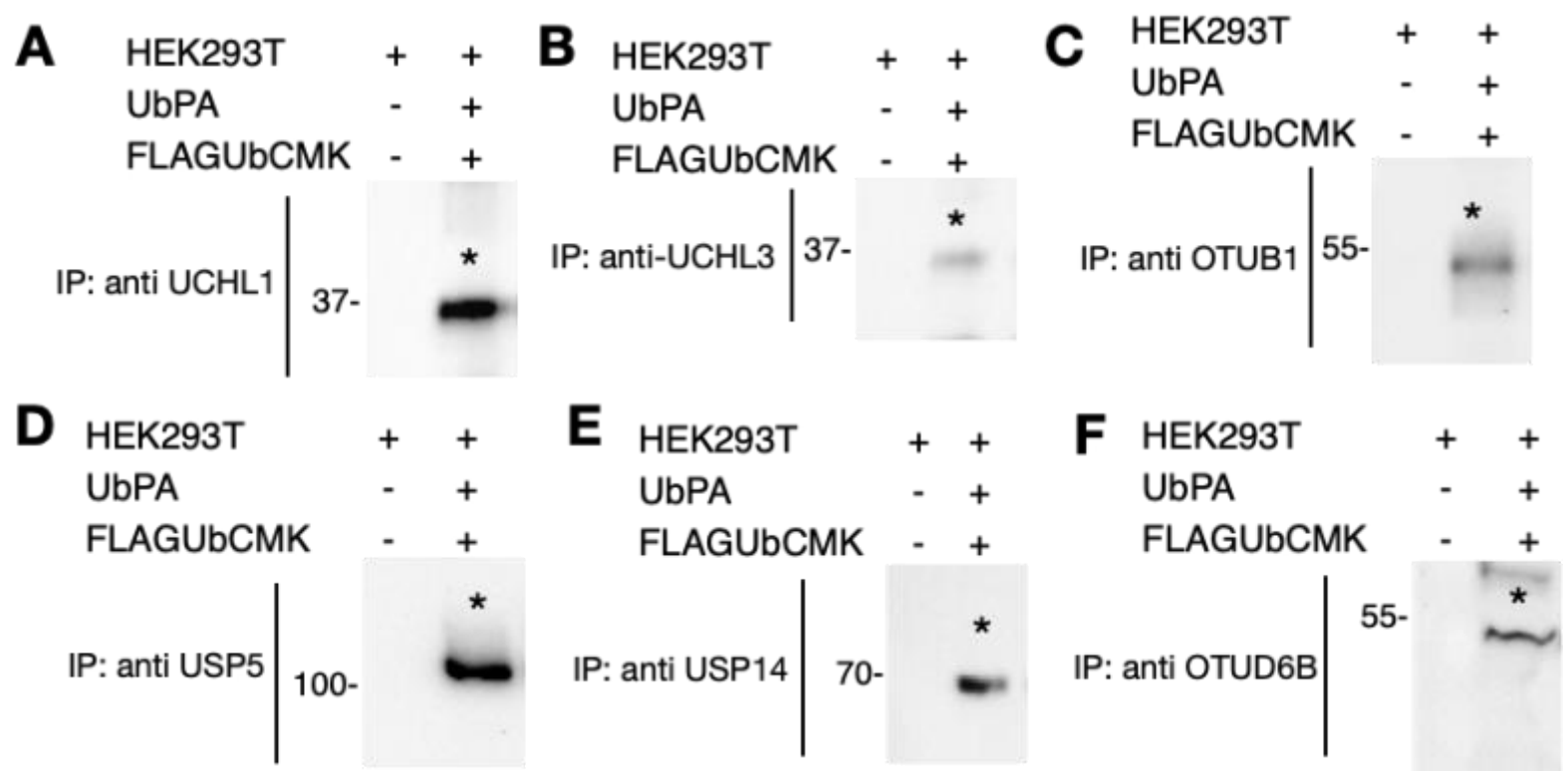

Figure S16. (**A-F**) Western blot validation of UCHL1, UCHL3, OTUB1, USP5, USP14, and OTUD6B in anti-FLAG immunoprecipitated samples from HEK293T cell lysates treated with UbPA for 12 h and then FLAGUbCMK or additional 12 h. Western blot detection was completed using corresponding antibodies for these enzymes. The asterisks represent the FLAGUbCMK-enzyme covalent complex.

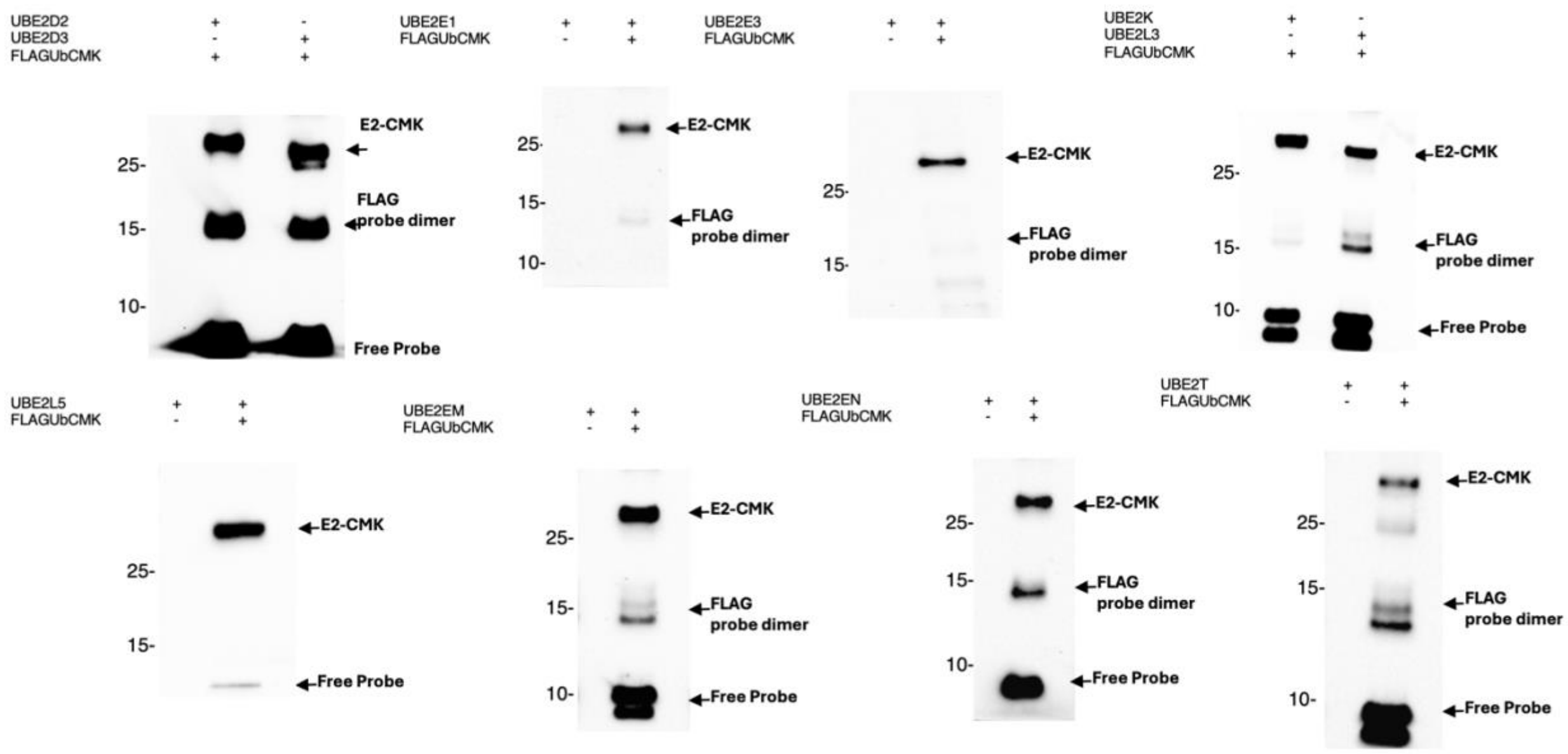

Figure S17. Whole gel images for Figure 6B

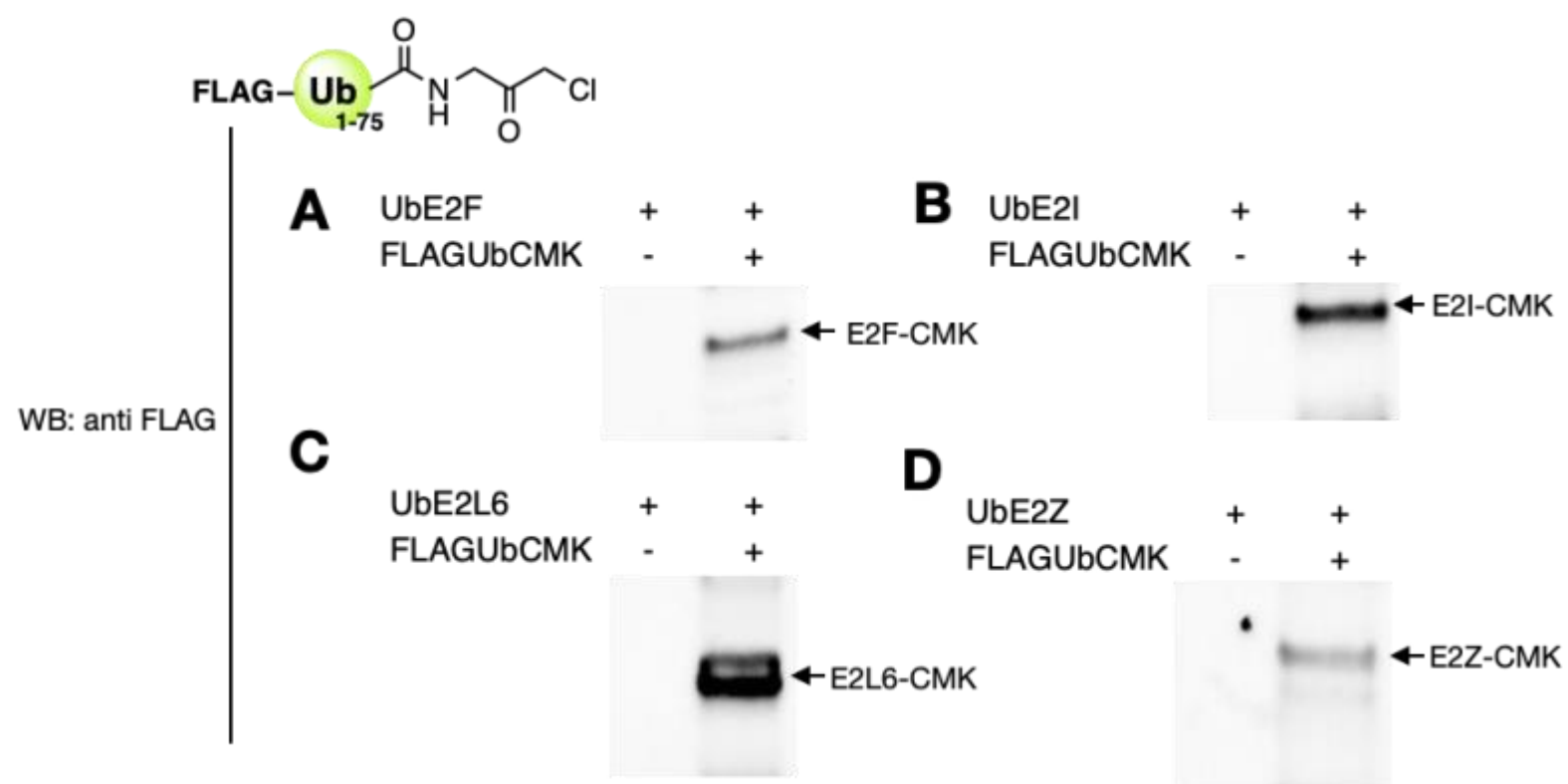

Figure S18. Covalent labeling of (A) UBE2F, (B) UBE2I, (C) UBE2L6, and (D) UBE2Z with FLAGUbCMK. Representative SDS–PAGE/anti-Flag immunoblots of recombinant UBE2F, UBE2I, UBE2L6, UBE2M, and UBE2Z incubated with FLAGUbCMK under standard assay conditions. All five E2s, previously reported to be unreactive toward UbDHA, were covalently labeled by FLAGUbCMK. The complex appeared as a discrete band as indicated (arrow) consistent with the MW of E2-CMK adducts.

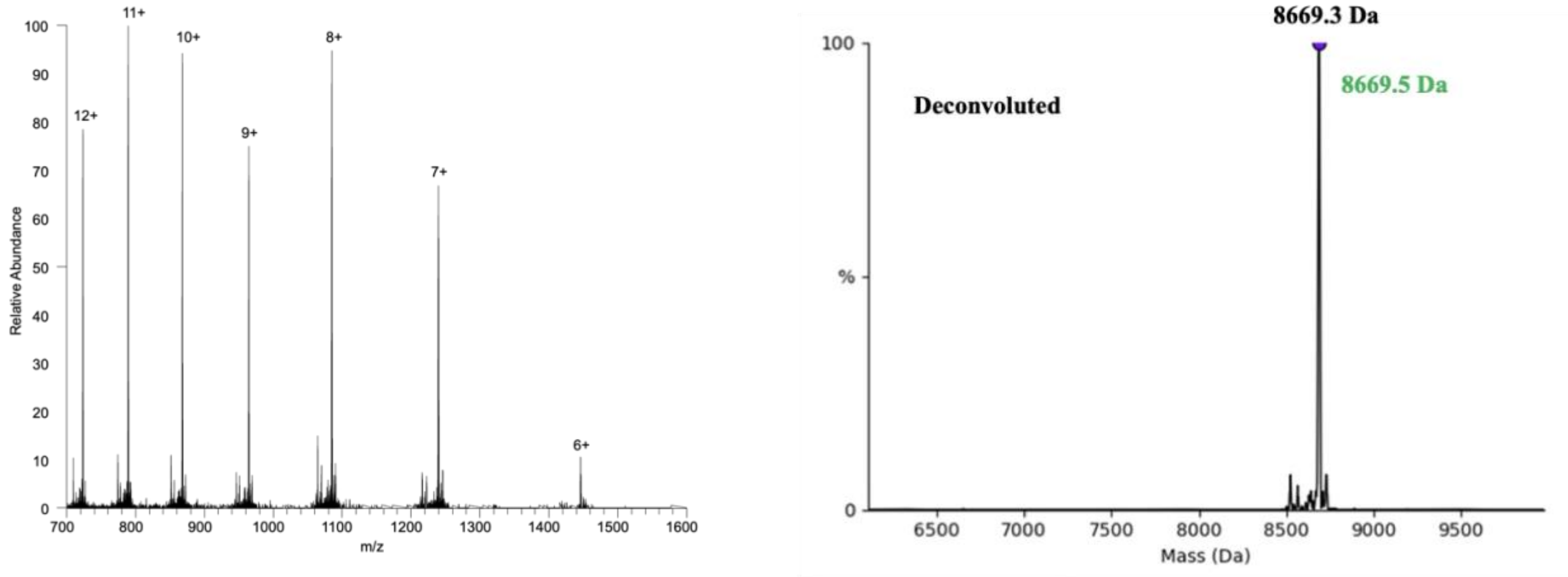

Figure S19. ESI-MS spectra and deconvoluted mass of UbCMK. The value in green indicates the actual theoretical mass of the probe.

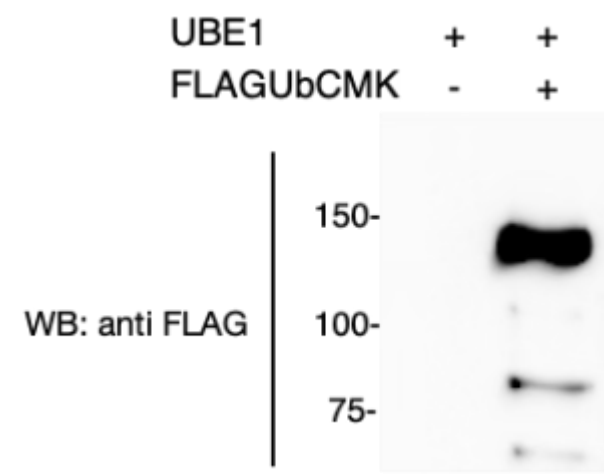

Figure S20. Reaction of FLAGUbCMK with UBE1. FLAGUbCMK was reacted with E1 enzyme. Reaction mixture was analyzed by SDS-PAGE followed by anti-FLAG western blot. The band represent the FLAGUbCMK-enzyme covalent complex.

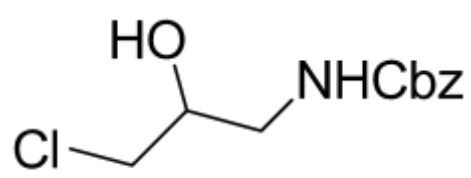

2

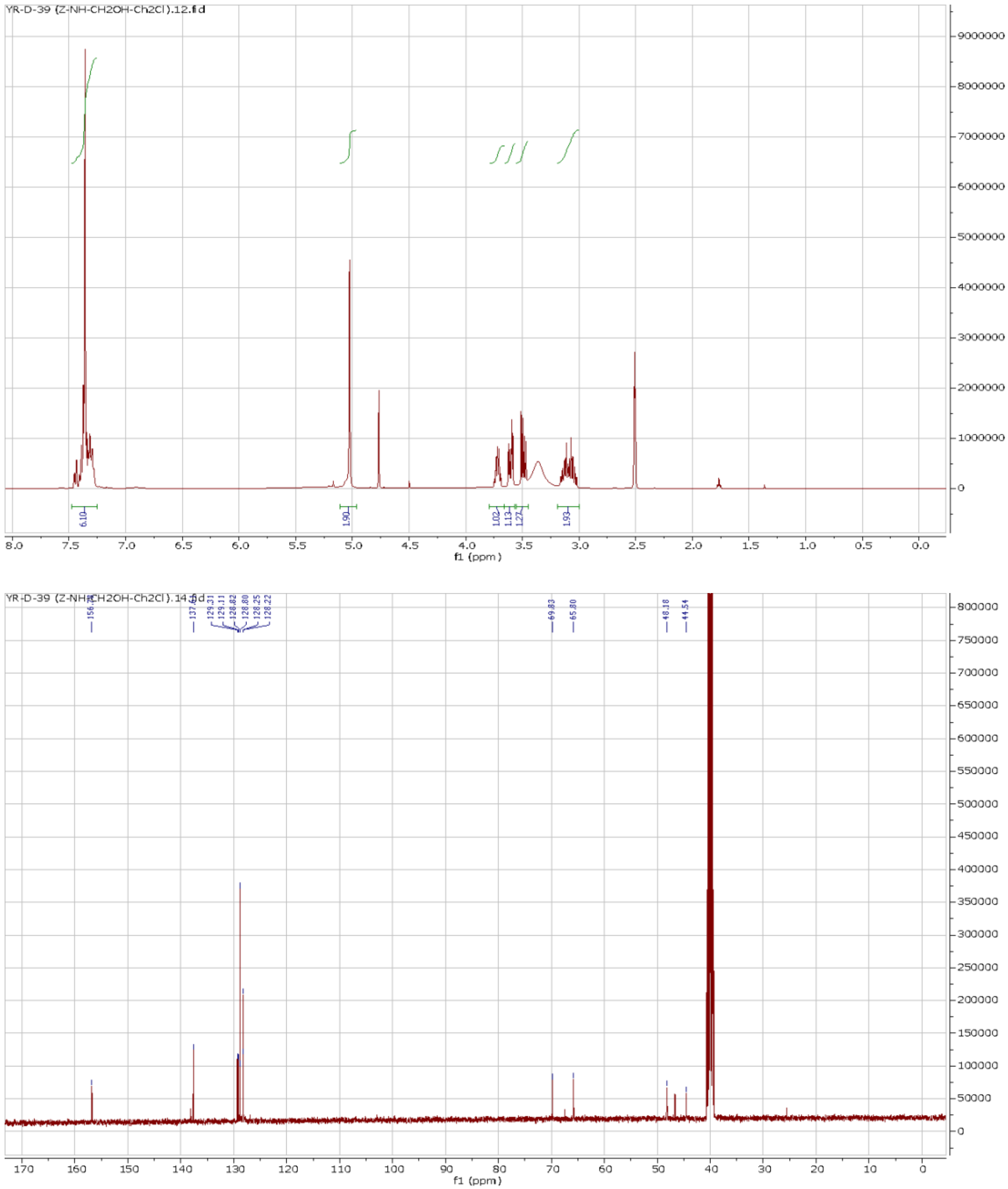

Figure S21. ^{1}H and ^{13}C NMR spectra of Compound 2.

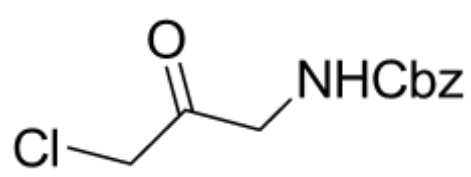

3

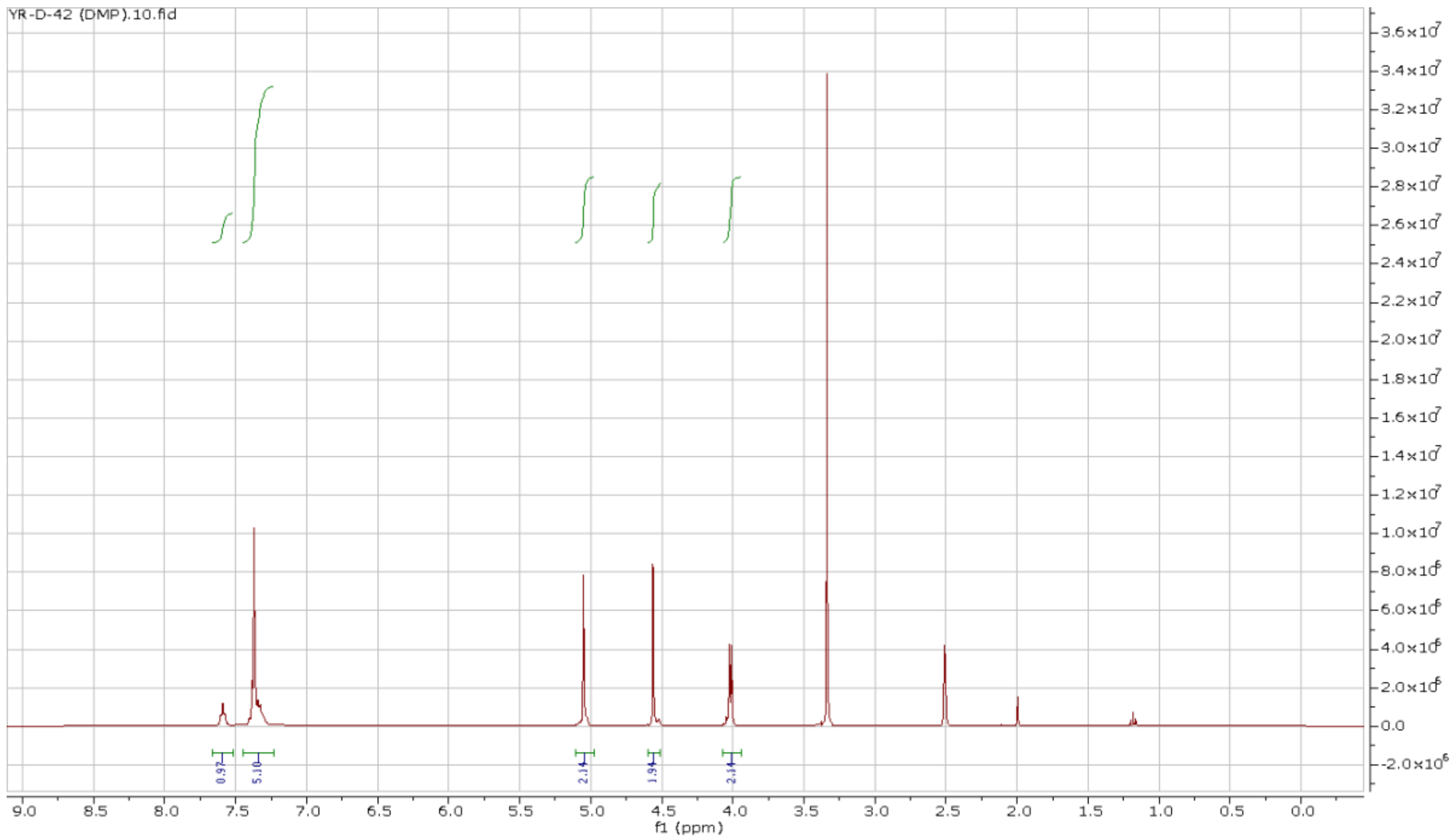

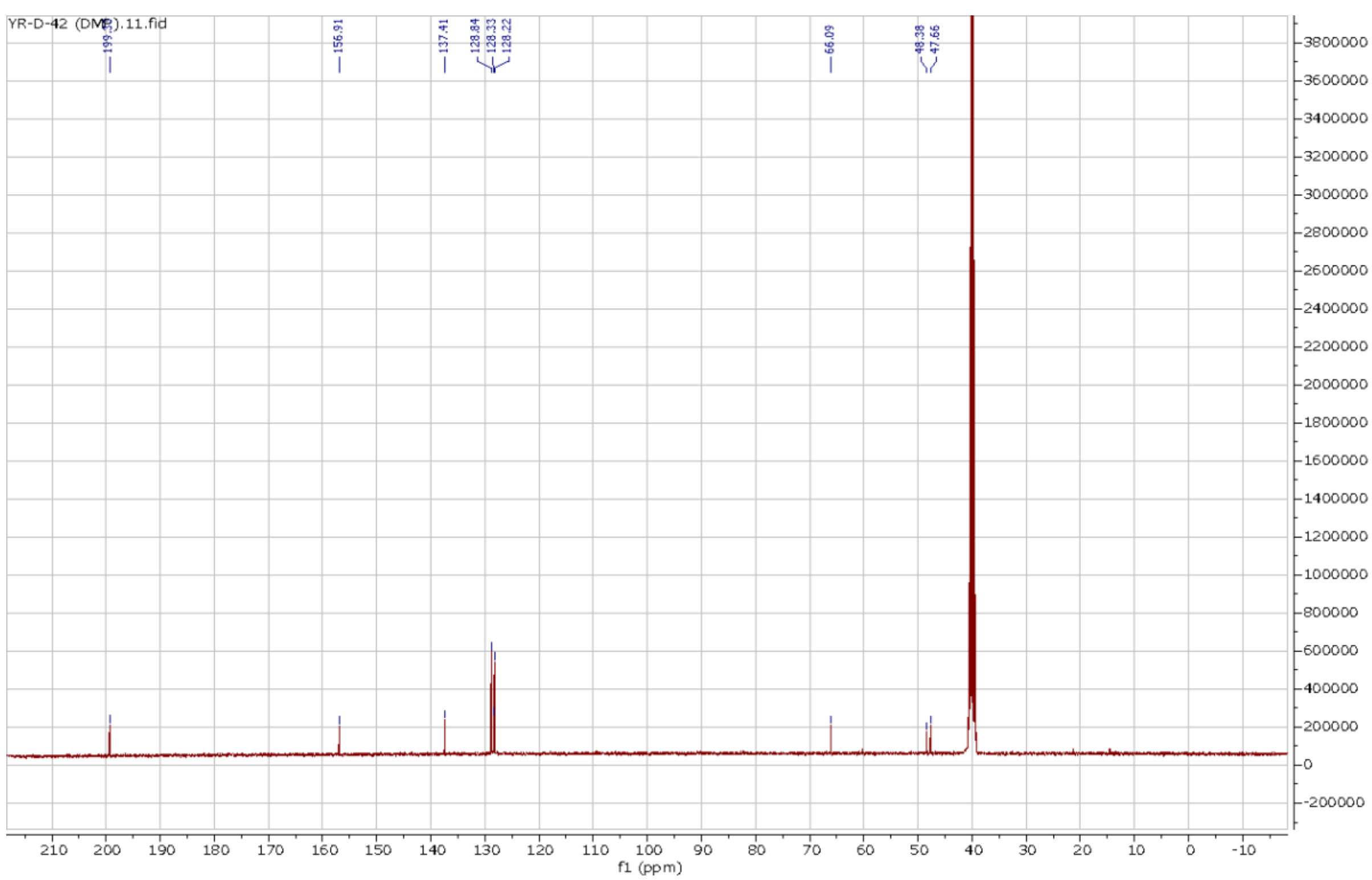

Figure S22. ^{1}H and ^{13}C NMR spectra of Compound 3.

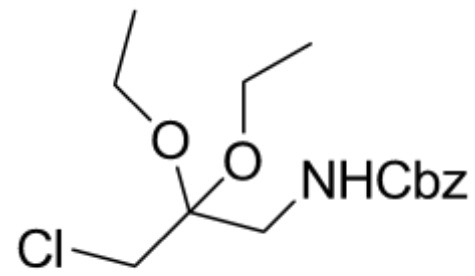

4

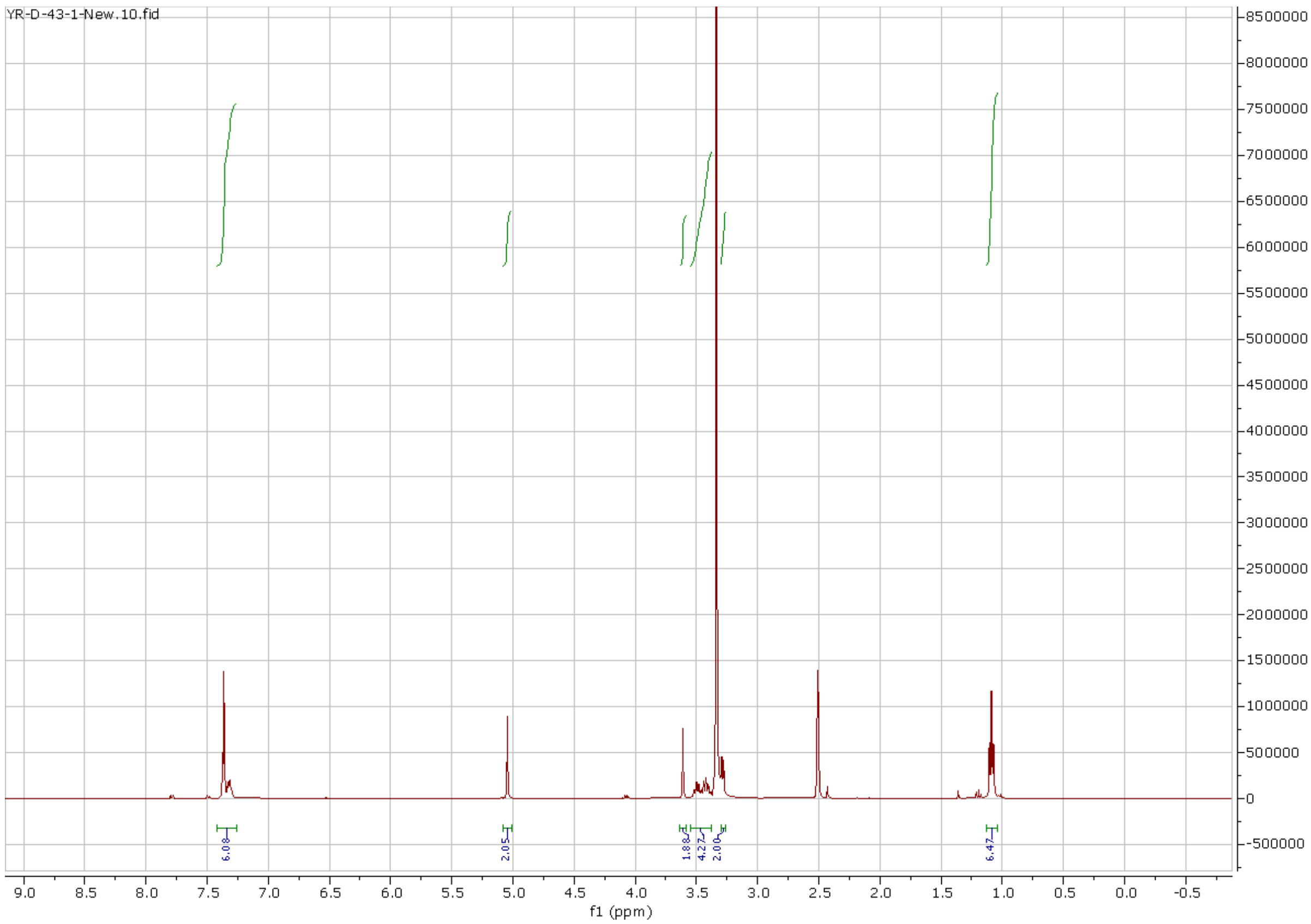

Figure S23. ^{1}H NMR spectra of Compound 4.

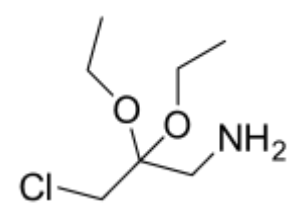

YR-D-53

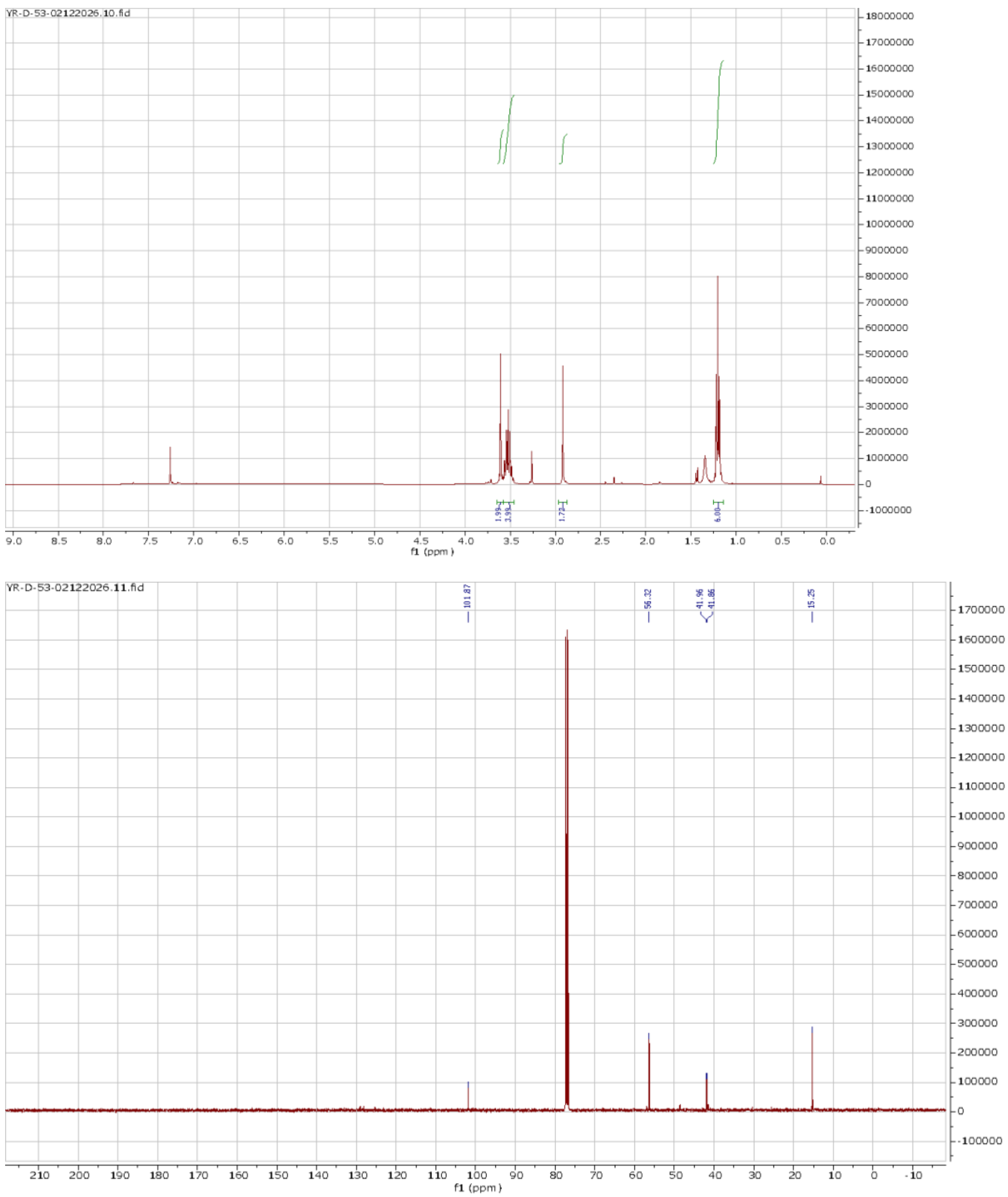

Figure S24. ^{1}H and ^{13}CNMR spectra of YR-D-53

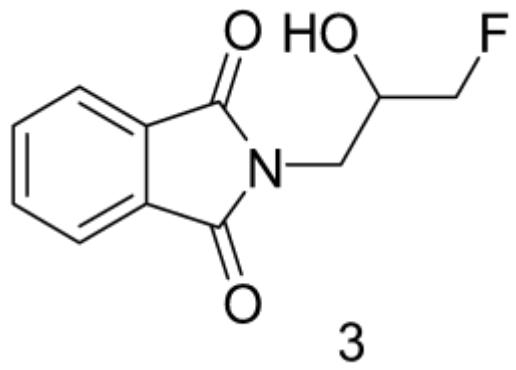

3

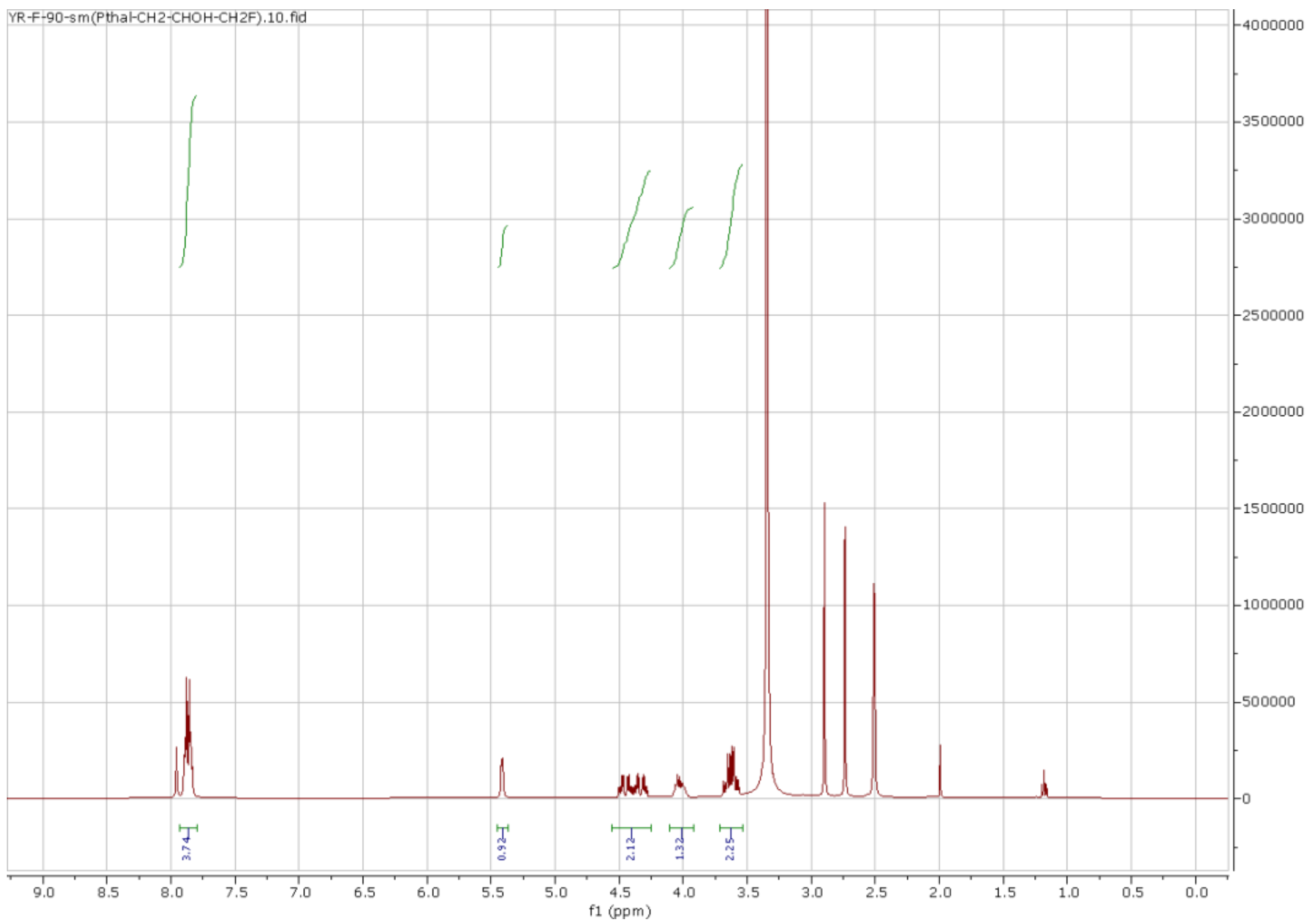

Figure S25. ^{1}H NMR spectra of Compound 3 (fluorinated compound).

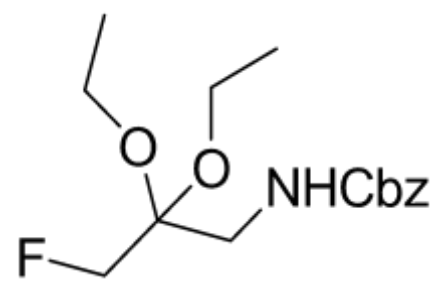

7

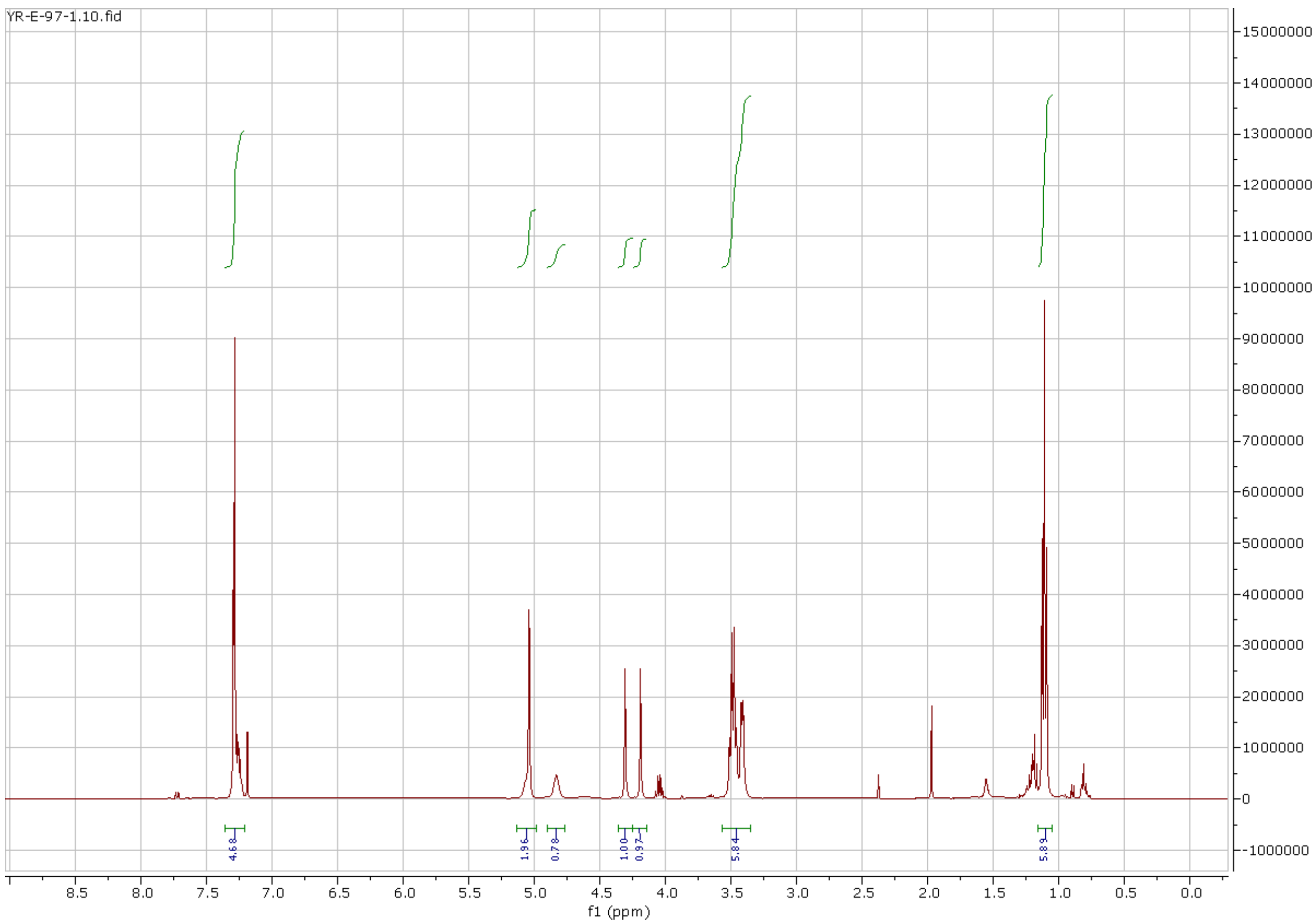

Figure S26. ^{1}H NMR spectra of Compound 7.

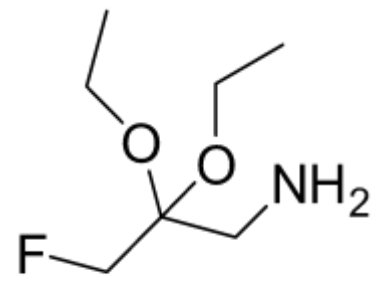

YR-E-99

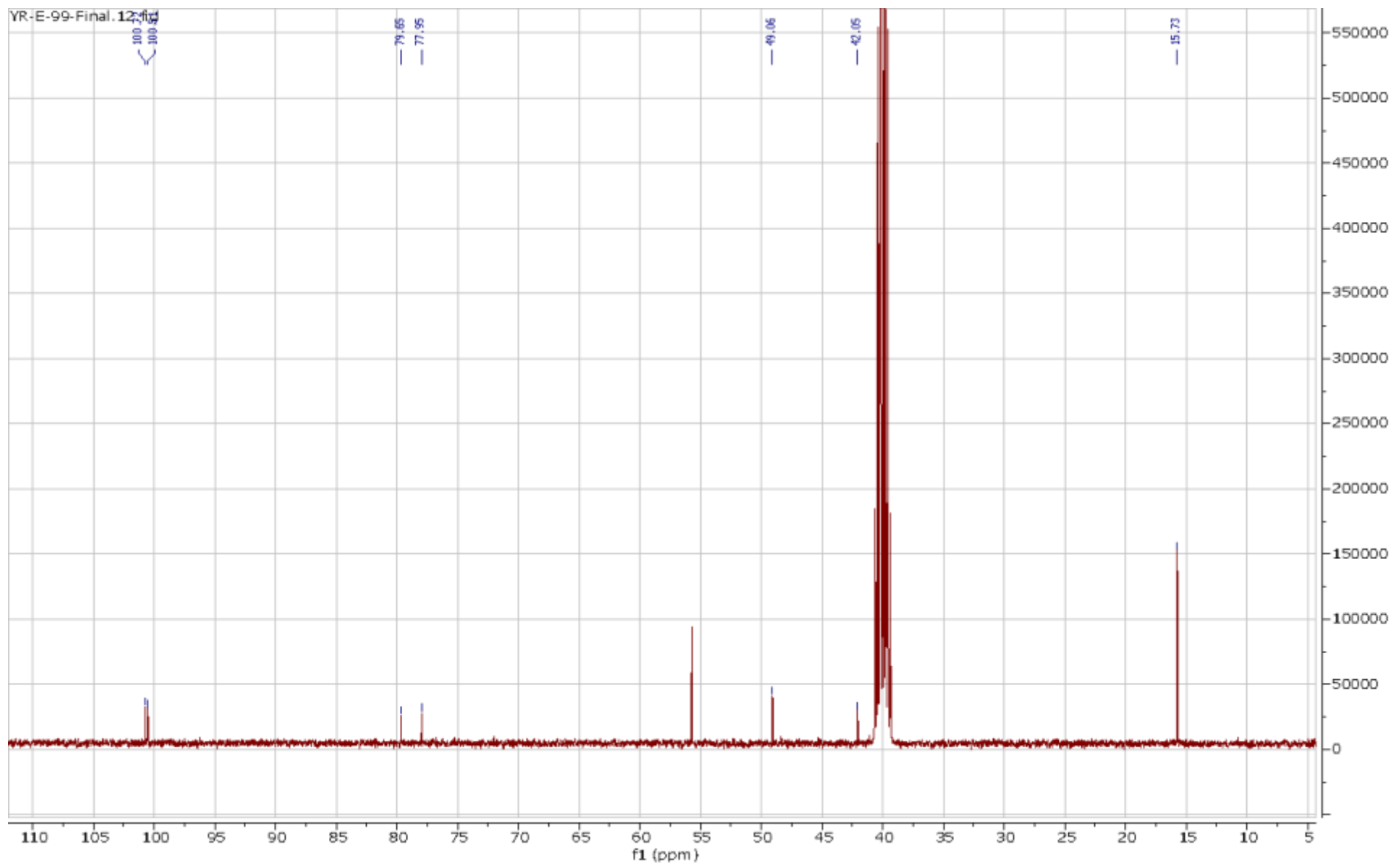

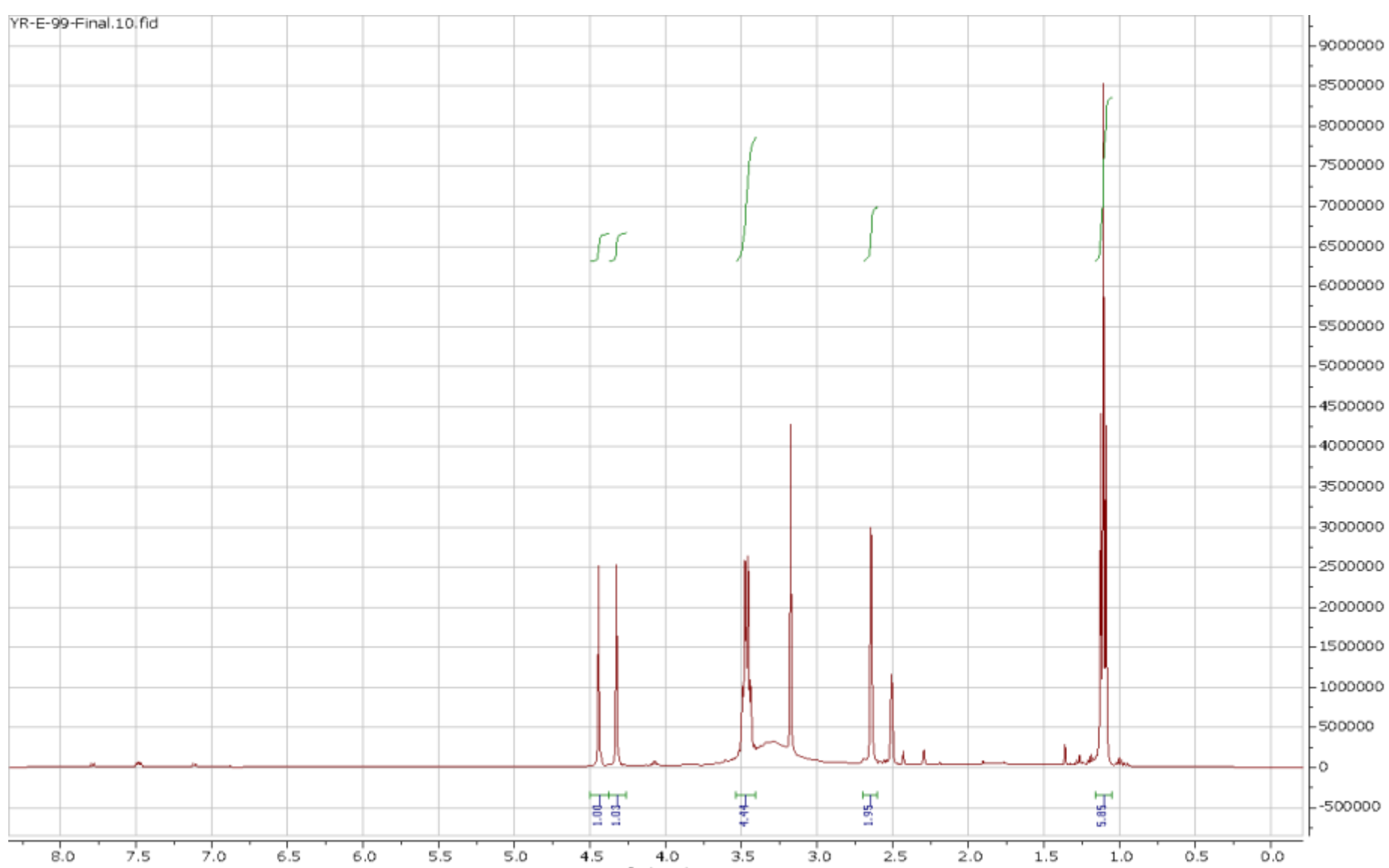

Figure S27. ^{19}F and ^{1}H NMR spectra of Compound YR-E-99.

Table S1. The molecular weight of Ub and synthesized Ub Activity-based probes

Protein	Observed mass (Da)	Theoretical mass (Da)
Ub_{1-75}-Cys-6×His	9433.4	9433.7
FLAG-Ub_{1-75}-Cys-6×His	10702.1	10702.0
UbPA	8543.6	8543.8
FLAGUbPA	9812.2	9812.1
FLAGUbFMK	9848.3	9848.5
FLAGUbCMK	9865.7	9865.7
UbCMK	8669.3	8669.5

Table S2A. The molecular weight of DUBs tested and formed DUB-Probe complex

Deubiquitinases	MW of Deubiquitinases	MW of DUB-Probe Complex
USP2 CD	~41 kDa	~51 kDa
USP5	~96 kDa	~106 kDa
USP7 CD	~41 kDa	~51 kDa
USP9X CD	~51 kDa	~61 kDa
USP10	~90 kDa	~109 kDa
USP14	~68 kDa	~78 kDa
UCHL1	~25 kDa	~35 kDa
UCHL3	~25 kDa	~35 kDa
GST Ataxin-3	~70 kDa	~80 kDa
OTUD6B	~37 kDa	~47 kDa

Table S2B. The molecular weight of E1, E3 enzymes tested and formed enzyme-probe complex

Enzymes	MW of Enzymes	MW of Enzyme-Probe Complex
UBE1	~118 kDa	~128 kDa

Table S3A. E2 detected in proteomics by Ub chloromethyl ketone probe in HEK293T cell lysate pretreated with UbPA.

Accession	Gene Symbol	Description
P51668	UBE2D1	Ub-conjugating enzyme E2 D1
P62837	UBE2D2	Ub-conjugating enzyme E2 D2
P61077	UBE2D3	Ub-conjugating enzyme E2 D3
Q9Y2X8	UBE2D4	Ub-conjugating enzyme E2 D4
P51965	UBE2E1	Ub-conjugating enzyme E2 E1
Q96LR5	UBE2E2	Ub-conjugating enzyme E2 E2
Q969T4	UBE2E3	Ub-conjugating enzyme E2 E3
P62253	UBE2G1	Ub-conjugating enzyme E2 G1
P60604	UBE2G2	Ub-conjugating enzyme E2 G2
P63279	UBE2I	SUMO-conjugating enzyme UBC9
P61086	UBE2K	Ub-conjugating enzyme E2 K
P68036	UBE2L3	Ub-conjugating enzyme E2 L3
A0A1B0GU54	UBE2L5	Ub-conjugating enzyme E2 L5
P61081	UBE2M	NEDD8-conjugating enzyme UBC12
P61088	UBE2N	Ub-conjugating enzyme E2 N
Q5JXB2	UBE2NL	UBE2N-like protein
Q9C0C9	UBE2O	Ub-conjugating enzyme E2 O
Q16763	UBE2S	Ub-conjugating enzyme E2 S
Q9NPD8	UBE2T	Ub-conjugating enzyme E2 T
Q13404	UBE2V1	Ub-conjugating enzyme variant 1
Q15819	UBE2V2	Ub-conjugating enzyme variant 2
Q9Y3C8	UFC1	UFM1-E2 conjugating enzyme

Table S3B. DUBs detected in proteomics by Ub chloromethyl ketone probe in HEK293T cell lysate pretreated with UbPA.

P09936	UCHL1	Ub carboxyl-terminal hydrolase isozyme L1
P15374	UCHL3	Ub carboxyl-terminal hydrolase isozyme L3
P45974	USP5	Ub specific protease 5
P54578	USP14	Ub specific protease 14
Q96FW1	OTUB1	Ub thioesterase OTUB1
Q8N6M0	OTUD6B	Deubiquitinase OTUD6B

Table S3C. E1s detected in proteomics by Ub chloromethyl ketone probe in HEK293T cell lysate pretreated with UbPA.

P22314	UBA1	Ub Activating Enzyme 1
Q9UBT2	UBA2	SUMO Activating Enzyme

Table S4. Other proteins identified in proteomics in HEK293T cell lysates pretreated with UbPA

Accession	Gene Symbol	Description
Q13867	BLMH	Bleomycin hydrolase
P68402	PAFAH1B2	platelet-activating factor acetylhydrolase Ib β-subunit
P55036	PSMD4	26S proteasome non-ATPase regulatory subunit
O75608	LYPLA1	Acyl-protein thioesterase
O00154	ACOT7	Cytosolic acyl co-enzyme thioester hydrolase
P07099	EPHX1	Microsomal epoxide hydrolase
P25786	PSMA1	Proteasome subunit alpha type-1
P67812	SEC11A	ER signal peptidase
P55786	NPEPPS	Puromycin sensitive aminopeptidase
Q9H4A4	RNPEP	Arginyl aminopeptidase
Q9NQH7	XPNPEP3	Mitochondrial aminopeptidase
P62979	RPS27A	Ub 40S ribosomal protein S27A
P62987	UBA52	Ub 40S ribosomal protein L40
Q10713	PMPCA	Mitochondrial metalloendopeptidase

The Gibbs Free Energy of Activation of four different chemical groups by a thiolate and formed transition state

Reactive warhead: Azapeptide Esters

Method: M06-2X/6-311+G(d,p), PCM(Water)
Electronic Energy/Hartree = -930.6009401
Gibbs Free Energy/Hartree = -930.472236

C	3.38630	-1.52146	-0.53557
C	2.61905	-0.38262	0.08100
H	4.44803	-1.37071	-0.34634
H	3.22383	-1.49498	-1.61479
H	3.07814	-2.49395	-0.15434
O	2.95350	0.78363	-0.01501
N	1.49238	-0.75121	0.76362
H	1.15873	-1.70373	0.74649
N	0.63210	0.19466	1.27050
C	-0.02989	1.06510	0.42323
O	-0.52541	2.09591	0.81673
O	-0.07472	0.61091	-0.82115
C	-0.89894	1.35952	-1.72400
H	0.92158	0.61866	2.14307
H	-1.93843	1.28790	-1.40072
H	-0.76429	0.89057	-2.69476
H	-0.57748	2.39965	-1.75569
S	-3.41152	-0.60503	0.41191
C	-2.21099	-1.65944	-0.49809
H	-2.61148	-2.66258	-0.65723
H	-1.27406	-1.75250	0.05530
H	-1.97176	-1.23212	-1.47425

Reactive warhead: Propargylamine

Method: M06-2X/6-311+G(d,p), PCM(Water)
Electronic Energy/Hartree = -762.8539333
Gibbs Free Energy/Hartree = -762.743990

C	0.09255	-2.39496	0.36496
C	1.09139	-1.32569	-0.01078
H	0.06853	-2.47836	1.45270
H	0.43417	-3.34964	-0.03792
H	-0.90868	-2.17519	-0.00606
O	2.26714	-1.38590	0.34183
N	0.60643	-0.31970	-0.76824
H	-0.41247	-0.21339	-0.88178
C	1.42300	0.83660	-1.08153
H	0.99743	1.32998	-1.95680
H	2.43055	0.50535	-1.33691
C	1.49215	1.79773	0.03127
C	1.52929	2.57939	0.94292
H	1.56422	3.27180	1.75236
S	-2.56853	0.37192	-0.60118
C	-2.16602	0.18257	1.18401
H	-2.50106	-0.78517	1.56280
H	-2.64568	0.96245	1.77752
H	-1.08679	0.25272	1.35091

Reactive warhead: Vinyl sulfone

Method: M06-2X/6-311+G(d,p), PCM(Water)
Electronic Energy/Hartree = -1351.9781429
Gibbs Free Energy/Hartree = -1351.813627

C	-4.44272	0.08355	-0.59341
C	-3.45625	-0.92849	-0.05948
H	-5.21383	0.24943	0.16013
H	-4.92390	-0.33406	-1.47898
H	-3.97004	1.03205	-0.84660
O	-3.81093	-2.05493	0.28510
N	-2.17685	-0.51475	0.00585
H	-1.92893	0.45317	-0.24740
C	-1.12702	-1.37548	0.51638
H	-1.36150	-2.40512	0.23025
H	-1.07531	-1.33924	1.60929
C	0.18765	-0.98579	-0.08682
C	1.27488	-0.74292	0.63145
S	2.75878	-0.22265	-0.17272
O	3.25158	0.96819	0.52706
O	2.51118	-0.12149	-1.61285
C	3.91769	-1.54417	0.10536
H	3.53432	-2.44112	-0.37686
H	4.03035	-1.68291	1.17906
H	4.85822	-1.22786	-0.34528
H	1.33603	-0.76616	1.71386
H	0.23170	-0.90047	-1.16956
S	-0.95803	2.48482	-0.43476
C	-0.35659	2.17789	1.27587
H	-0.57951	3.02615	1.92527
H	0.72216	2.01063	1.28767
H	-0.83437	1.29231	1.70722

Reactive warhead: Chloromethylketone

Method: M06-2X/6-311+G(d,p), PCM(Water)
Electronic Energy/Hartree = -1298.94259
Gibbs Free Energy/Hartree = -1298.817648

C	-4.16322	-0.51574	-0.82195
C	-3.08156	-0.26039	0.19892
H	-5.04097	-0.90907	-0.30912
H	-3.85619	-1.20956	-1.60292
H	-4.43785	0.43750	-1.27642
O	-3.28335	0.41731	1.20192
N	-1.87762	-0.81325	-0.05501
H	-1.71398	-1.33597	-0.90448
C	-0.74385	-0.57791	0.80125
H	-0.84885	-1.10183	1.75965
H	-0.63904	0.48884	1.01301
C	0.51940	-1.06663	0.12412
O	0.49528	-1.70426	-0.89723
C	1.78272	-0.74797	0.89518
H	1.81194	0.31636	1.11821
Cl	3.25814	-1.16712	-0.00697
H	1.78656	-1.31544	1.82692
S	0.74854	2.09579	-1.03287
C	1.20377	2.78189	0.61125
H	1.02082	3.85687	0.65723
H	2.26197	2.61755	0.83078
H	0.62150	2.32089	1.41590

Transition state formed by Azapeptide Esters

Method: M06-2X/6-311+G(d,p), PCM(Water)
Electronic Energy/Hartree = -930.5739381
Gibbs Free Energy/Hartree = -930.443926

C	4.01513	0.29690	-0.53723
C	2.72686	-0.30918	-0.03878
H	4.64790	-0.50213	-0.92631
H	4.52931	0.75395	0.30829
H	3.86056	1.04493	-1.31371
O	2.69270	-1.10352	0.89395
N	1.61166	0.07030	-0.70581
H	1.69351	0.54530	-1.59241
N	0.35878	-0.53057	-0.46307
C	-0.45811	0.37611	0.35522
O	-0.16693	0.68855	1.50601
O	-1.01375	1.27279	-0.51536
C	-1.78969	2.28855	0.10036
H	0.53637	-1.35778	0.10175
H	-2.61497	1.83332	0.65562
H	-2.17807	2.90783	-0.70558
H	-1.18297	2.88975	0.77867
S	-2.25469	-1.15699	0.52214
C	-2.85679	-1.02745	-1.19548
H	-3.60854	-1.79130	-1.40047
H	-2.02264	-1.15685	-1.88698
H	-3.29895	-0.04534	-1.37221

Transition state formed by Propargylamine

Method: M06-2X/6-311+G(d,p), PCM(Water)
Electronic Energy/Hartree = -762.8088201
Gibbs Free Energy/Hartree = -762.698707

C	3.09725	-1.29382	0.64538
C	2.43380	-0.08959	0.01839
H	3.83810	-0.94613	1.36658
H	3.62252	-1.84577	-0.13499
H	2.39211	-1.95645	1.14518
O	3.08492	0.75307	-0.59831
N	1.10367	0.01146	0.18371
H	0.57665	-0.74514	0.60029
C	0.32825	1.09130	-0.41436
H	0.31588	0.97465	-1.50408
H	0.81928	2.04005	-0.18410
C	-1.06255	1.12723	0.12078
C	-1.92833	1.83623	0.69398
H	-1.91730	2.84038	1.08989
S	-1.73150	-0.95766	-0.47005
C	-3.39526	-0.81654	0.20235
H	-3.58190	-1.55600	0.98140
H	-4.15294	-0.91555	-0.57544
H	-3.45238	0.19886	0.63741

Transition state formed by vinyl sulfone

Method: M06-2X/6-311+G(d,p), PCM(Water)
Electronic Energy/Hartree = -1351.9639311
Gibbs Free Energy/Hartree = -1351.797262

C	-4.56203	-0.36162	-0.70212
C	-3.28181	-0.88336	-0.09340
H	-5.36743	-1.05857	-0.46900
H	-4.44221	-0.33830	-1.78645
H	-4.82958	0.63325	-0.34903
O	-2.88428	-2.02299	-0.31961
N	-2.61582	-0.02876	0.71331
H	-2.92143	0.93200	0.76272
C	-1.31886	-0.34784	1.27626
H	-1.32180	-1.41335	1.51827
H	-1.19169	0.21342	2.20237
C	-0.15315	-0.08174	0.34376
C	1.12203	-0.19061	0.86652
S	2.46460	-0.52897	-0.13667
O	3.67634	0.12809	0.39688
O	2.12541	-0.26657	-1.54966
C	2.80269	-2.28568	-0.03682
H	1.92757	-2.81935	-0.40297
H	3.00454	-2.53472	1.00355
H	3.67392	-2.48664	-0.66017
H	1.37071	-0.05511	1.91163
H	-0.30045	-0.42004	-0.67613
S	-0.70761	2.13686	-0.22492
C	0.94925	2.84638	-0.06072
H	1.37986	3.09700	-1.03081
H	1.59621	2.10265	0.42497
H	0.93224	3.74142	0.56153

Transition state formed by chloromethylketone

Method: M06-2X/6-311+G(d,p), PCM(Water)
Electronic Energy/Hartree = -1298.9373685
Gibbs Free Energy/Hartree = -1298.810401

C	-4.05346	-0.33291	-0.86091
C	-3.00801	-0.17099	0.21763
H	-4.97893	-0.68205	-0.40300
H	-3.75123	-1.02508	-1.64552
H	-4.24533	0.64653	-1.30269
O	-3.23633	0.47231	1.24061
N	-1.82376	-0.76416	-0.01364
H	-1.64329	-1.23300	-0.89178
C	-0.68129	-0.60615	0.85145
H	-0.68800	-1.37372	1.63934
H	-0.70443	0.37081	1.33121
C	0.59302	-0.81633	0.02864
O	0.56368	-1.58119	-0.93831
C	1.83683	-0.67680	0.89756
H	1.83384	0.24375	1.47456
Cl	3.35879	-0.73687	-0.03832
H	1.86481	-1.53064	1.57876
S	0.63417	1.46823	-0.94213
C	1.19387	2.52983	0.43705
H	1.03425	3.58301	0.20083
H	2.25817	2.38605	0.64006
H	0.63581	2.30965	1.35240
